# An algorithmic system for arabic fake news detection using neural networks and transformer embeddings with class weighting

**DOI:** 10.1038/s41598-026-45653-4

**Published:** 2026-04-13

**Authors:** Mohamed Saad, Samir Abdelrazek, Islam R. Abdelmaksoud

**Affiliations:** Faculty of Computers and Information, Department of Information Systems, Mansoura University, 35516 Mansoura, Egypt

**Keywords:** Fake news, Arabic, BERT, SMOTE, NN, ClassWeight-ArabicBERT-NN, Computer science, Information technology

## Abstract

Modern communication technologies have increased the speed of news dissemination. Information can now reach wide audiences almost instantly. At the same time, these technologies can reduce traditional verification mechanisms, contributing to the accelerated spread of false information and posing potential societal and political challenges. While research on fake news detection has increased, Arabic remains under-explored due to limited datasets and the language’s morphological and semantic complexity. We compiled a multi-source Arabic news dataset consisting of 7,474 articles. The dataset was meticulously validated, achieving high labeling quality (Fleiss’ Kappa = 0.80). Preliminary experiments with baseline algorithms indicated that the neural network (NN) consistently outperformed the other models. This supported its selection as the core classifier in the proposed framework. The proposed system integrates an NN classifier with CAMeLBERT embeddings for semantic feature extraction. A comprehensive comparison was conducted with other prominent Arabic Transformers, including AraBERT, AraELECTRA, and MARBERTv2. We then evaluated multiple imbalance-handling techniques, including class weighting, undersampling, oversampling, and SMOTE. Performance was assessed under different configurations, highlighting the benefits of combining contextual embeddings with resampling strategies. Experimental results indicated that the CAMeLBERT-based neural network with class weighting achieves competitive performance across the evaluated configurations, attaining an F1-score of 96.19%, accuracy of 95.52%, precision of 95.48%, and recall of 96.90% in Arabic fake news detection. These findings indicate that the proposed model provides a reliable basis for automated Arabic fact-checking systems. In addition to predictive performance, the study strengthens methodological rigor through the integration of LIME and SHAP-based interpretability analyses. Future work will focus on assessing cross-domain generalization and investigating the feasibility of real-time deployment.

## Introduction

The proliferation of fake news has become a major global concern, largely due to the widespread use of smartphones and social media. It often spreads misinformation with serious social and political consequences^1^. Such news can take various forms, including fabrications, scams, and deceptive tactics^2^.

Although extensive research has been conducted on fake news detection in English, studies on Arabic content remain limited^3^. Most current approaches for Arabic fake news detection rely on classical machine learning, data-level resampling techniques (e.g., undersampling, oversampling, SMOTE), or modern Transformer-based DNN models. While these methods can improve performance, they may introduce limitations such as synthetic noise (in SMOTE), overfitting risk (in random oversampling), or significant data loss (in random under-sampling)^4^. Previous studies often report results using a limited set of evaluation metrics or concentrate on a single model, which restricts their generalization and practical applicability. These limitations indicate a critical research gap: there is a limited systematic investigation of algorithm-level strategies, such as Class Weighting, for handling class imbalance in Arabic fake news datasets. This gap hinders the development of robust and reliable detectors for real-world Arabic applications^5^.

To overcome these challenges, we propose a resource-efficient hybrid framework for Arabic fake news detection. It focuses on mitigating class imbalance and ensuring effective model performance. The framework integrates three main components. First, we applied Class Weighting approach to address data imbalance. This algorithm-level method observed better performance than data-level resampling techniques while minimizing synthetic noise and data loss. Second, CAMeLBERT serves as a fixed feature extractor. It provides rich contextual embeddings and performs better than traditional methods like TF-IDF and Bag-of-Words^6^. We also used a Transformer-specific preprocessing pipeline, which performs better than traditional normalization methods (e.g., stemming and stop word removal)^7^. Third, a deep neural network (DNN) classifier is employed to capture non-linear relationships in the dense embedding space. It offers greater expressive power than linear classifiers^8^. The strategic integration of Combining Class Weighting with CAMeLBERT and the DNN creates an efficient architecture. It effectively addresses class imbalance, captures rich contextual information, and remains suitable for real-world, resource-constrained applications.

Furthermore, we perform a rigorous comparative analysis of four state-of-the-art Arabic Transformer architectures (CAMeLBERT, AraBERT^9^, MARBERTv2^10^, and AraELECTRA^11^).This study advances Arabic fake news detection by making the following contributions:

- **Systematic Evaluation of Algorithm-level Imbalance Handling:** We conduct a comprehensive empirical analysis of algorithm-level Class Weighting, highlighting its robustness and advantages over multiple data-level imbalance handling techniques.
- **Transformer Selection Insights for Arabic Fake News Detection:** We provide empirical insights into the suitability of different state-of-the-art Arabic Transformer models, identifying the most effective feature extractor for Arabic fake news detection under realistic experimental settings.
- **High-Quality Large-Scale Dataset:** We compile and meticulously validate a multi-source Arabic news dataset comprising **7,474 articles**. We ensure high annotation reliability with a Fleiss’ Kappa^12^ score of 0.80, providing a robust and diverse foundation for training and evaluation.
- **Interpretability and Linguistic Insights:** We provide a comprehensive LIME and SHAP analysis that mitigates the ”black-box” nature of the DNN classifier and delivers evidence-based explanations revealing linguistically meaningful patterns influencing the model’s decision-making process for Arabic fake news detection.

The remainder of this article is organized as follows: Section “Related work” reviews the challenges in detecting fake news on social media and discusses related work. Section "Proposed hybrid framework" describes the proposed Arabic fake news detection framework based on CAMeLBERT, DNN, and Class Weighting. Section “Experimental results” presents detailed insights into the system’s performance, discussing the experimental results and Model Interpretability Analysis. Section “Discussion” provides a comprehensive discussion of the findings, highlighting the practical implications, real-world applications, and inherent limitations of the proposed approach. Finally, Section "Conclusion and future work" concludes the study and outlines directions for future research and advancements in Arabic fake news detection.

## Related work

Fake news detection has become an important research topic in recent years. Most studies focus on English-language content, while Arabic remains an underexplored domain. Researchers have applied a range of methods to detect and classify fake news, from classical machine learning to modern deep learning and NLP approaches. However, several challenges remain, especially in handling imbalanced datasets and evaluating models comprehensively across multiple metrics.

In order to categorize fake news, researchers frequently use binary classifiers to determine whether the information is real or fake. Alternatively, some studies address this challenge using multi-class classification, clustering, or regression approaches. Recently, three types of strategies have been proposed to identify fake news^13^. The first strategy examines trends in the distribution of news across the network using user interactions, shares, and comments. The second strategy focuses on user profiles, tracking user activity through posting history, reactions, comments, messages, and social connections such as location, followers, friends, and groups. Finally, the third strategy, which falls under the category of syntactic and semantic-based approaches, is based on an analysis of the news content. These strategies highlight current approaches and their limitations, motivating a review of prior studies in this field. Consequently, the subsequent sections present pertinent previous work categorized by the type of analysis (e.g., content-based, user-based, etc.) that informed our model development.

M. Granik and V. Mesyura^14^ developed an approach to identify fake news utilizing a Naive Bayes classifier. Their dataset was compiled from three mainstream political news sources and three substantial left and right-leaning Facebook pages. They achieved a classification accuracy of approximately 74%. Although this work was pioneering, it also reveals the limitations of classical machine learning approaches when applied to complex textual tasks.

M.L. Della et al.^15^ initially introduced a method that combined features from both social context and the news content itself, achieving an accuracy of 78.8%, which outperformed earlier approaches. They later extended this method into a Facebook Messenger chatbot for practical verification, which improved the accuracy to 81.7% in detecting fake news. The final dataset included 15,500 posts collected from 32 pages, supported by over 2.3 million likes from more than 900,000 unique users.

M. S. Looijenga^16^ proposed a model for detecting counterfeit content using N-gram analysis along with various feature extraction techniques. The study evaluated six different machine learning algorithms. The best results were obtained using a unigram feature set with both a Decision Tree (DT) and a Linear Support Vector Machine (LSVM) classifier. This combination achieved a maximum accuracy of 92% and an F1-score of 0.88.

P. Kaur et al.^17^ conducted a comparative study on detecting fake news using various machine learning approaches. The evaluation considered three classifiers: Naïve Bayes, neural networks, and support vector machines (SVMs). The study highlighted the importance of data normalization as a critical step in preparing the dataset for classification. While Naïve Bayes achieved an accuracy of 96.08%, the more advanced classifiers, NNs and SVMs, performed exceptionally well, reaching an accuracy of 99.90%.

Safaya et al.^18^ developed a hybrid BERT-CNN model that leverages BERT’s contextual understanding alongside the feature extraction strengths of convolutional neural networks. Their results showed that this hybrid approach substantially outperformed BERT alone as well as five other benchmark models in their comparison. Notably, the system achieved a strong F1-score of 0.897 on Arabic-language content, demonstrating the effectiveness of hybrid models for this task.

Singh et al.^19^ proposed an attention-based LSTM network that utilizes tweet text along with thirteen linguistic and user-related features to differentiate between rumor and non-rumor tweets. The model showed strong performance, achieving an F1-score of 0.88, outperforming several benchmark models.

Mangal and Sharma^20^ applied two deep learning models, CNN and LSTM, to a dataset of 1,000 news articles for classification as fake or real. Their model achieved an accuracy of 91.1%. Additionally, their approach incorporated the cosine similarity index as part of the classification process.

Rohit et al.^21^ proposed FakeBERT, an architecture that combines multiple single-layer deep CNN blocks with different kernel sizes and filters to extract multi-scale features from BERT embeddings. They evaluated the model on a dataset of content from the 2016 U.S. presidential election, showing that this approach effectively captures linguistic ambiguity and semantic nuances in deceptive news, achieving a peak accuracy of 98.90%. While FakeBERT demonstrates the strength of hybrid CNN-Transformer models, our study extends this approach to Arabic, incorporating class-balancing techniques and interpretability layers that are often lacking in high-accuracy ”black-box” models.

Junaid and Rashid^22^ employed deep learning and NLP to effectively detect bogus news on social media. To detect fake news, textual data can be evaluated using bidirectional transformer designs such as BERT. The suggested system outperforms existing benchmarks for detecting false news propagated during the 2016 U.S. elections, including cutting-edge techniques on a real-world dataset. In comparison to the present cutting-edge model, this system has 90% fewer network parameters and is 75% lower in size.

Koru et al.^23^ developed a pre-trained BERT model that was subsequently fine-tuned. They also evaluated extended versions of the model incorporating Bi-LSTM and convolutional neural network layers under both frozen and unfrozen parameter settings. The dataset was labeled and preprocessed using Zemberek, a natural language processing tool tailored for the Turkish language. The analysis employed ensemble learning methods along with Bag-of-Words (BoW), TF-IDF, and Word2Vec vectorization techniques to detect fake news. Model performance was compared across seven benchmark datasets and achieved a range from 90% to 94% of the highest accuracy.

Hussain and Aslam^24^ focused on detecting hate speech on Twitter, particularly content aimed at women and immigrants. They tested several text embeddings–TF-IDF, CBOW, and GloVe–alongside Random Forest and SVM classifiers. Among these, SVM combined with GloVe produced the strongest results, achieving 84.79% accuracy and an F1-score of 84.93% in distinguishing targeted from non-targeted posts. The study demonstrates the effectiveness of using embeddings for hate speech detection, though it was restricted to English-language tweets.

Othman et al.^25^ introduced a hybrid deep learning approach that integrates AraBERT embeddings with a 2D-CNN for detecting Arabic fake news. The model was evaluated on the ANS, AraNews, and Covid19Fakes datasets, achieving 71% accuracy on ANS and F1-scores reaching 80.09%. These results highlight the potential of combining Transformer embeddings with CNNs for effective Arabic news classification.

Hussain Ali^3^ conducted a study on Arabic fake news detection using the AFND dataset, which includes over 600,000 articles labeled as credible, not credible, or undecided. The approach employed AraBERT along with preprocessing steps such as stopword removal, stemming, normalization, and diacritic handling. The model achieved 92.3% accuracy and a macro F1-score of 72%, outperforming traditional machine learning approaches, showing the effectiveness of transformer-based models and domain-specific preprocessing.

Turki et al.^26^ explored Arabic fake news detection using WaraBERT, a hybrid feature extraction method that combines word-level tokenization with two AraBERT variants. Experiments on the AFND and AraNews datasets (123,219 records) showed that WaraBERT-V1 with BiLSTM achieved 93.83% accuracy on AFND, while WaraBERT-V2 reached 81.25% on AraNews, outperforming traditional approaches such as WLT, TF-IDF, and AraBERT. These results highlight the effectiveness of hybrid contextual embeddings in capturing nuanced Arabic semantics for fake news detection. However, the computational demands of hybrid BiLSTM-Transformer models often limit scalability in real-world applications, a challenge our study addresses with a streamlined CAMeLBERT-DNN framework.

A recent survey by Touahri and Mazroui (2024)reviewed machine learning techniques for detecting fake news in Arabic and highlighted that while many studies address preprocessing, feature extraction, and model selection, there is limited emphasis on handling class imbalance explicitly^5^.

In summary, most existing studies on fake news detection have focused on English datasets, with only a few addressing Arabic content^3,26^. Although many approaches achieve high accuracy, they often overlook a major challenge: class imbalance, which is common in real-world datasets where instances of fake news are much fewer than legitimate ones. Advanced deep learning techniques have considerably improved Arabic fake news detection. Notable performance gains using transformer-based models and other DNN architectures have been reported in^3,18,21,26^. Moreover, most studies attempting to address class imbalance rely mainly on data-level resampling techniques, such as SMOTE or oversampling, which can introduce noisy synthetic examples. Undersampling, in contrast, may discard valuable data from the majority class. Therefore, there remains a clear need for approaches that better manage class imbalance and provide robust performance^5,27^.

Furthermore, earlier works often report limited evaluation metrics or focus on a single ”black-box” model, which restricts the understanding of robustness and hinders adoption in critical applications^14,15,19,20^. To address these limitations, our study proposes a three-tier framework: (1) CAMeLBERT is used for high-fidelity Arabic contextual representations, (2) algorithm-level Class Weighting ensures predictive fairness without distorting the data, and (3) a dual-layer interpretability analysis using SHAP and LIME provides insights into model decisions. By comparing multiple baseline algorithms with our proposed framework, this approach offers a robust, transparent, and scalable solution for maintaining information integrity in the Arabic digital space. Table 1 summarizes existing algorithms, datasets, and evaluation metrics.

**Table 1:** Previous automated systems for fake news detection.

Reference	Year	Algorithm(s) used	Dataset	Evaluation metrics
M. Granik and V. Mesyura^14^	2017	Naive Bayes classifier	Collection of Facebook news articles	Accuracy =74%
M. L. Della Vedova^15^	2018	Content-based, logistic regression, harmonic Boolean label crowdsourcing	15,500 postings	Accuracy = 81.7%
M. S. Looijenga^16^	2018	N-gram, LSVM, DT	A total of 613,033 tweets were sent in the Dutch election.	Accuracy = 92.00%, F1-score = 0.88
P. Kaur et al.^17^	2019	Naïve Bayes, NN, and SVM	Online fake news	Accuracy = 99.90%
Safaya et al.^18^	2020	BERT, CNN	Sets of tweets (Offensive (positive) or non-offensive (negative))	F1-score = 0.851
Singh et al.^19^	2020	Attention-based LSTM	Publicly available Pheme dataset	F1-score = 0.88
Mangal and Sharma^20^	2020	CNN, LSTM	1000 news on social media	Accuracy = 91.10%
Rohit et al.^21^	2021	CNN, BERT	Fake news propagated during the 2016 U.S. election	Accuracy = 98.90%
Junaid and Rashid^22^	2024	NLP, sequential neural networks, BERT	20718 labeled instances	Accuracy = 99.9%, F1-score = 99.9%
Koru et al.^23^	2024	BERT, Bi-LSTM, CNN (frozen & unfrozen)	Fake & real news from Twitter (Turkish, preprocessed with Zemberek)	Accuracy = 90%–94%
Hussain and Aslam^24^.	2024	Random Forest, SVM + (TF-IDF, CBOW, GloVE embeddings)	Twitter (Hate speech against women and immigrants) 12,200 tweets	Accuracy = 84.79%, F1-score = 84.93%
Othman et al.^25^	2024	Hybrid AraBERT + 2D-CNN	ANS	Accuracy 71%
H.A.Bahri^3^	2025	AraBERT (Transformer-based model), compared with traditional ML methods (e.g., SVM, Naïve Bayes, Logistic Regression)	AFND (600,000+Arabic news articles; categories: credible, not credible, undecided)	Accuracy: 92.3%Macro F1-score:72%
Turki et al.^26^	2025	WaraBERT-V1 (AraBERT + wordlevel tokenization + BiLSTM) WaraBERT-V2 (hybrid contextual features) Compared with WLT, TF-IDF, AraBERT	AFND (606,912 records) AraNews (123,219 entries)	AFND (WaraBERT-V1) : 93.83% accuracyAraNews (WaraBERT-V2) : 81.25% accuracy

## Proposed hybrid framework

NLP relies on a variety of artificial intelligence models to understand human language. Among these, BERT^9^ is widely used. It is based on transformer architectures, which effectively capture linguistic and semantic nuances^28^. In addition to transformer-based models, general neural networks are also commonly employed in deep learning. They consist of multiple layers and weighted connections, which are gradually updated during training to learn patterns from large datasets^29^. This section outlines the key components involved in constructing the proposed framework.

The proposed framework introduces a hybrid approach for Arabic text classification. It leverages CAMeLBERT embeddings as a feature extractor, combined with a feed-forward DNN classifier. A Transformer-specific preprocessing step ensures robust handling of diacritics, elongations, and orthographic variations^30^. Additionally, class imbalance is addressed using the Class Weighting algorithm.

**Fig. 1:**
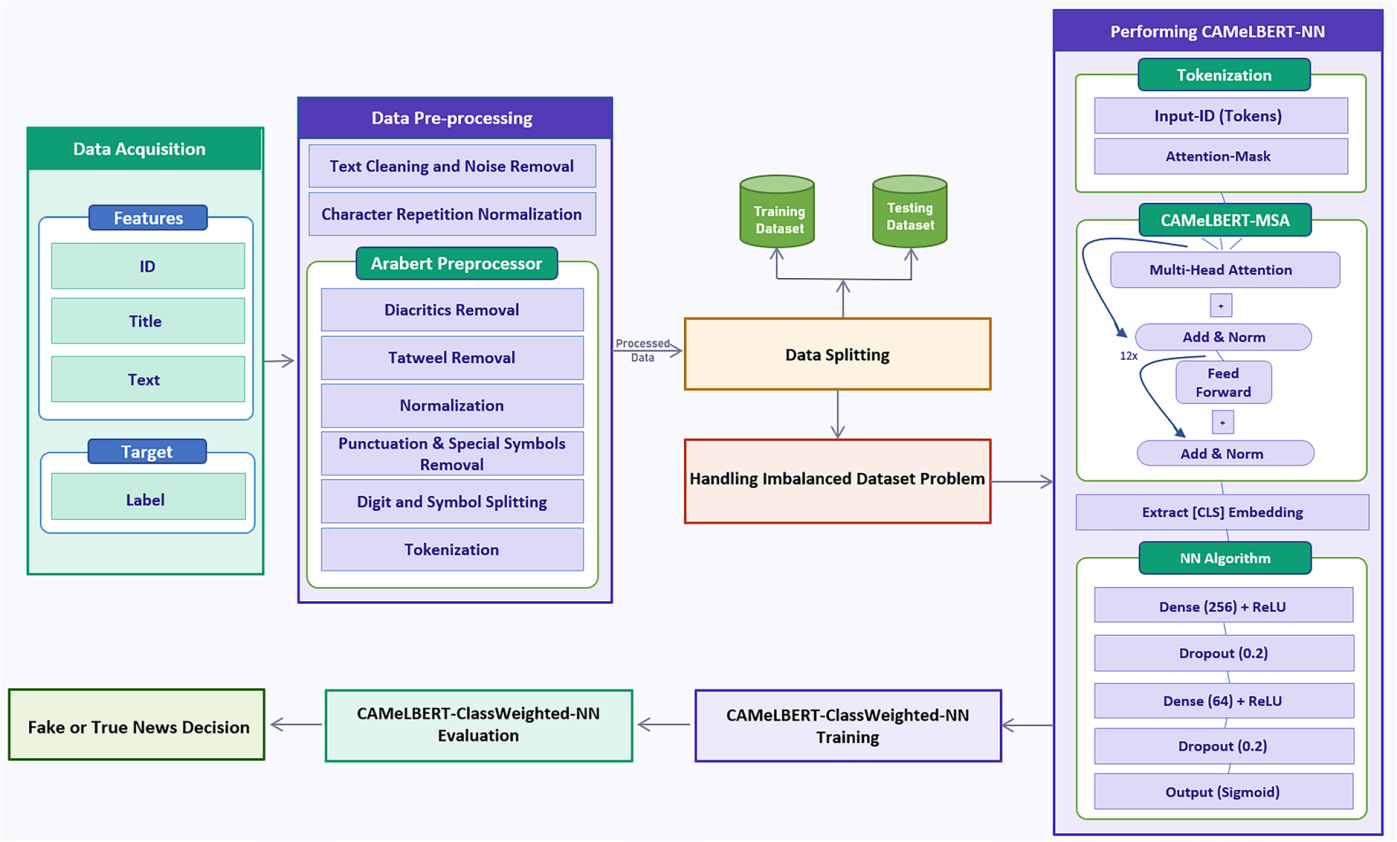
Block diagram of the proposed fake news detection system.

The design choice of this hybrid framework is motivated by several factors. First, using CAMeLBERT as a fixed feature extractor provides rich semantic embeddings without the high computational cost of fully fine-tuning Transformer-based approaches. This makes it suitable for large-scale or real-world applications with limited computational resources. Second, combining these embeddings with a DNN classifier allows the model to capture complex non-linear relationships that traditional linear classifiers may miss^8^. Third, Class Weighting is used to address class imbalance and avoid potential noise or overfitting introduced by data-level resampling techniques such as SMOTE or oversampling. Compared to conventional ML pipelines or full fine-tuning of Transformers, this design provides a balanced approach. It ensures robust contextual representation, computational efficiency, and effective handling of underrepresented classes. As a result, the framework is reproducible and performs well for Arabic fake news detection. Moreover, this hybrid design enhances scalability and reusability. By decoupling feature extraction from classifier training, CAMeLBERT embeddings can be reused across different datasets or related tasks. This avoids full model retraining and enables efficient experimentation while reducing computational overhead. All preprocessing steps, model architecture, and hyperparameters are comprehensively documented, ensuring full reproducibility of the framework and facilitating its adaptation for future research. The subsequent subsections describe dataset acquisition, data preprocessing, data splitting, model implementation, training, performance evaluation, and the generation of classification reports indicating whether the news is fake or real. Figure 1 presents a block diagram summarizing these components, each of which is discussed in detail in the following subsections.

### Data acquisition

The dataset was carefully compiled through a multi-source collection strategy to include news articles spanning multiple categories, such as political, economic, and social news, ensuring diversity and supporting generalizability of the proposed model. Articles were gathered from three primary categories of sources to ensure reliability:

(i) Trusted news sources (~45%): Reliable news articles were collected from established and reputable Arabic news agencies, including well-known regional media platforms. These sources provided verified, high-quality content used to represent legitimate news.
(ii) Unverified online content (~30%): Additional articles were collected from public online spaces such as blogs, open Facebook pages, and user-generated posts. These samples typically exhibit higher rates of misinformation and were manually labeled by the research team following a rigorous verification protocol. To capture the characteristics of social media dissemination, this subset reflects informal linguistic styles and rapid propagation patterns typical of user-generated online content. Each article was cross-referenced against official news agencies and reputable Arabic fact-checking platforms, such as Fatabyyano^31^ and the Arab Fact-Checkers Network (AFCN)^32^. The criteria for labeling an article as ’fake’ included the presence of fabricated claims, lack of credible attribution, or direct contradictions with verified reports from official national and international statements.
  To validate the labeling quality and objectivity, a challenging subset of 500 samples was independently double-coded by the three members of the research team. The inter-rater agreement was subsequently calculated using Fleiss’ Kappa^12^, yielding a high score of $\kappa$ = 0.80, which confirms the substantial agreement and consistency of our applied criteria
(iii) External benchmark datasets (~25%): To enhance coverage and reduce domain dependency, part of the dataset was enriched using samples adapted from well-known fake-news benchmark datasets, such as WELFake^33^ and LIAR^34^. Relevant articles were translated into Arabic.

The translation process was carried out using the Google Translate API (via the deep-translator package in Python^35^) to ensure high efficiency. Crucially, the translated content was subsequently subjected to a rigorous human post-editing and quality assurance phase by a native Arabic speaker to guarantee linguistic accuracy and, most importantly, preserve the context and deceptive linguistic nuances essential for the fake news detection task.

The dataset consists of 7,474 Arabic news articles spanning the period from 2015 to 2025, covering political, economic, and social topics. Political news dominates the dataset, with international political news (~40%) and national political news (~15%). Economic and social news collectively account for (~25%), while the remaining (~20%) is distributed across other related categories, with a higher proportion of political news, while maintaining coverage of other relevant topics. The dataset primarily focuses on Modern Standard Arabic (MSA), ensuring geographical inclusivity and linguistic consistency across the Arab world. It also includes elements of ’White Arabic’, a simplified semi-formal style prevalent in digital journalism, to better reflect contemporary online content. This strategic focus avoids regional dialectal bias, ensuring that the model remains robust and generalizable across diverse Arabic-speaking populations while maintaining the authenticity required to detect misinformation in social media contexts.

The dataset is structured in tabular form, where each record includes an identifier of the news sample (Id), the news title (the headline of the news article), the full text (the main body of the article), and the corresponding label, which serves as the target variable in the classification task (real or fake). The dataset is moderately imbalanced, with 4,359 samples (58.32%) labeled as real and 3,115 samples (41.68%) labeled as fake, reflecting the natural skew typically observed in real-world news content.

### Data pre-processing

After constructing the dataset, the collected raw Arabic texts contained various inconsistencies, including diacritics, elongations (tatweel), emojis, special characters, and noise from user-generated content, which can increase data sparsity and hinder model effectiveness. To ensure robust representation, we adopted a comprehensive preprocessing pipeline specifically tailored for modern Arabic Transformer models.

We utilized the AraBERT Preprocessor, a specialized tool designed to handle the linguistic complexities of Arabic text^36^. This approach was preferred over traditional methods (stemming or stop-word removal) as it preserves the morphological and contextual integrity vital for transformer-based embeddings.

The preprocessing pipeline consisted of the following sequential steps:

- Cleaning and Noise Removal: Non-informative elements such as hashtags, user mentions, and hyperlinks were removed using regular expressions (regex). This technique enabled automated identification and elimination of such elements to ensure that only semantically meaningful content is fed into the model.
- Character-Level Normalization: Informal Arabic writing often contains character-level noise, including exaggerated character repetition for emphasis (e.g., elongated forms such as jameeeel or halooo), excessive whitespace, and spelling irregularities or incorrect characters. These patterns were normalized to their standard forms to reduce noise and improve model robustness.

Following these initial cleaning steps, the AraBERT preprocessor was applied to perform advanced linguistic standardization, ensuring the text is fully compatible with the WordPiece tokenization scheme. The preprocessor performs the following integrated tasks:

- **Removing diacritics:** Arabic texts often include diacritics, which can increase data sparsity. Removing them ensures consistency in word representation.
- **Removing elongations:** Some words in the Arabic language include unnecessary elongation characters for stylistic purposes. These were normalized to their base forms.
- **Normalization of characters:** Different forms of certain Arabic letters (such as ”A” with different diacritical marks) were unified into a single representation to reduce vocabulary size.
- **Sub-word decomposition (WordPiece):** Rare or unseen words are decomposed into sub-words using the WordPiece algorithm, ensuring better vocabulary coverage and representation.
- **Removing unwanted symbols:** Special characters, emojis, URLs, and punctuation not relevant to the classification task were removed.
  By integrating the AraBERT preprocessor with the CAMeLBERT tokenizer, the dataset was transformed into a clean, standardized format. This synergy ensures that the hybrid model receives high-quality linguistic inputs, directly contributing to the overall accuracy and robustness of the fake news detection system.

### Data splitting

The dataset was divided into distinct subsets using the stratified random sampling technique to ensure proportional representation of both ”real” and ”fake” news classes in all partitions, mitigating the risk associated with the observed class imbalance. The hold-out cross-validation method was applied, splitting the dataset into training and testing partitions. An 80%/20% train-test split was adopted for training and optimizing the downstream classifier and for testing its predictive performance on unseen data, respectively^37^. A fixed random state of 44 was adopted for all experiments. All data splitting parameters are detailed in Table 2.

### Applying CAMeLBERT-classweighting-NN system

The core contribution of this study is the proposed hybrid framework, which combines the strong contextual representation capabilities of the CAMeLBERT-base model specifically pre-trained for Modern Standard Arabic (MSA version^6^) (used as a fixed feature extractor) with a sophisticated Deep neural network classifier. This architecture is specifically enhanced by the integration of the Class Weighting technique during training. This combination is designed to effectively leverage rich contextual embeddings while simultaneously addressing the challenges posed by class imbalance and the inherent complexity of Arabic fake news detection.

#### Handling the imbalanced dataset problem using class weighting

After splitting the data, we encountered a significant imbalance in our dataset, with a disproportionate distribution of class labels for fake and true news, where the number of real news instances greatly exceeds that of fake news instances. The presence of this imbalance reduces the effectiveness of the system, as it leads to unstable classification and a tendency to bias toward the incorrect class during prediction. Therefore, it is essential to address this issue to avoid misleading results^38,39^. To address this, both data-level strategies (e.g., SMOTE, oversampling, undersampling) and algorithm-level approaches (e.g., Class Weighting) can be employed.

Data-level resampling techniques are commonly used to mitigate class imbalance, but each method has inherent limitations. Random oversampling duplicates minority class instances, which can lead to overfitting and reduce the model’s generalizability. Random undersampling removes instances from the majority class, potentially discarding valuable information and negatively impacting overall performance^40,41^. SMOTE (Synthetic Minority Oversampling Technique) generates synthetic samples for the minority class, but it may introduce noise or create unrealistic instances that do not reflect the true data distribution^42^. In contrast, algorithm-level approaches such as Class Weighting adjust the loss function to account for imbalance without altering the original data^43^. Unlike oversampling or undersampling techniques, Class Weighting doesn’t require modifying the data or generating synthetic samples. This helps prevent issues such as overfitting and information loss, thereby offering a more robust solution for training models on imbalanced datasets.

In this study, we focus on Class Weighting as an algorithm-level solution because it proved to be the most effective strategy in our experiments. To implement this, we applied Class Weighting in the loss function of transformer-based neural networks. We systematically compared this approach with several data-level balancing techniques, including SMOTE, random oversampling, and random undersampling, across the same baselines (NN, Transformer-NN variants, and conventional models). Experimental results demonstrate that Class Weighting consistently outperforms all other methods across multiple evaluation metrics. This confirms that Class Weighting provides a robust and reliable framework for handling imbalanced datasets in Arabic fake news detection.

#### The CAMeLBERT feature extractor

The feature extraction layer of our proposed system utilizes the CAMeLBERT model. CAMeLBERT is a state-of-the-art Arabic Transformer model built upon the architecture of BERT^6^. BERT pre-training relies primarily on the Masked Language Modeling (MLM) objective, which enables the model to learn rich, bidirectional contextual representations by predicting randomly masked tokens within a sentence^44^. This bidirectionality allows the resulting embeddings to capture deeper semantic and syntactic dependencies, making them well suited for downstream classification tasks^45^.

Selecting the appropriate Arabic BERT variant is essential for ensuring optimal alignment with the linguistic characteristics of our dataset. CAMeLBERT provides several pretrained versions trained on different Arabic corpora, including Mix, MSA, DA (Dialectal Arabic), and CA (Classical Arabic)^46^. This study employs the CAMeLBERT-base MSA model, which is trained exclusively on Modern Standard Arabic. This selection maximizes compatibility with our dataset composed entirely of MSA news articles, ensuring that the extracted embeddings remain linguistically consistent and minimizing representation noise. Among the available MSA variants (full, half, quarter, etc.), the base model offers the best balance between representational quality and computational efficiency, making it suitable for both experimentation and potential real-world deployment. All CAMeLBERT variants are pretrained on Arabic corpora only, unlike multilingual BERT models, ensuring more stable and semantically coherent embeddings for Arabic texts.

**Fig. 2:**
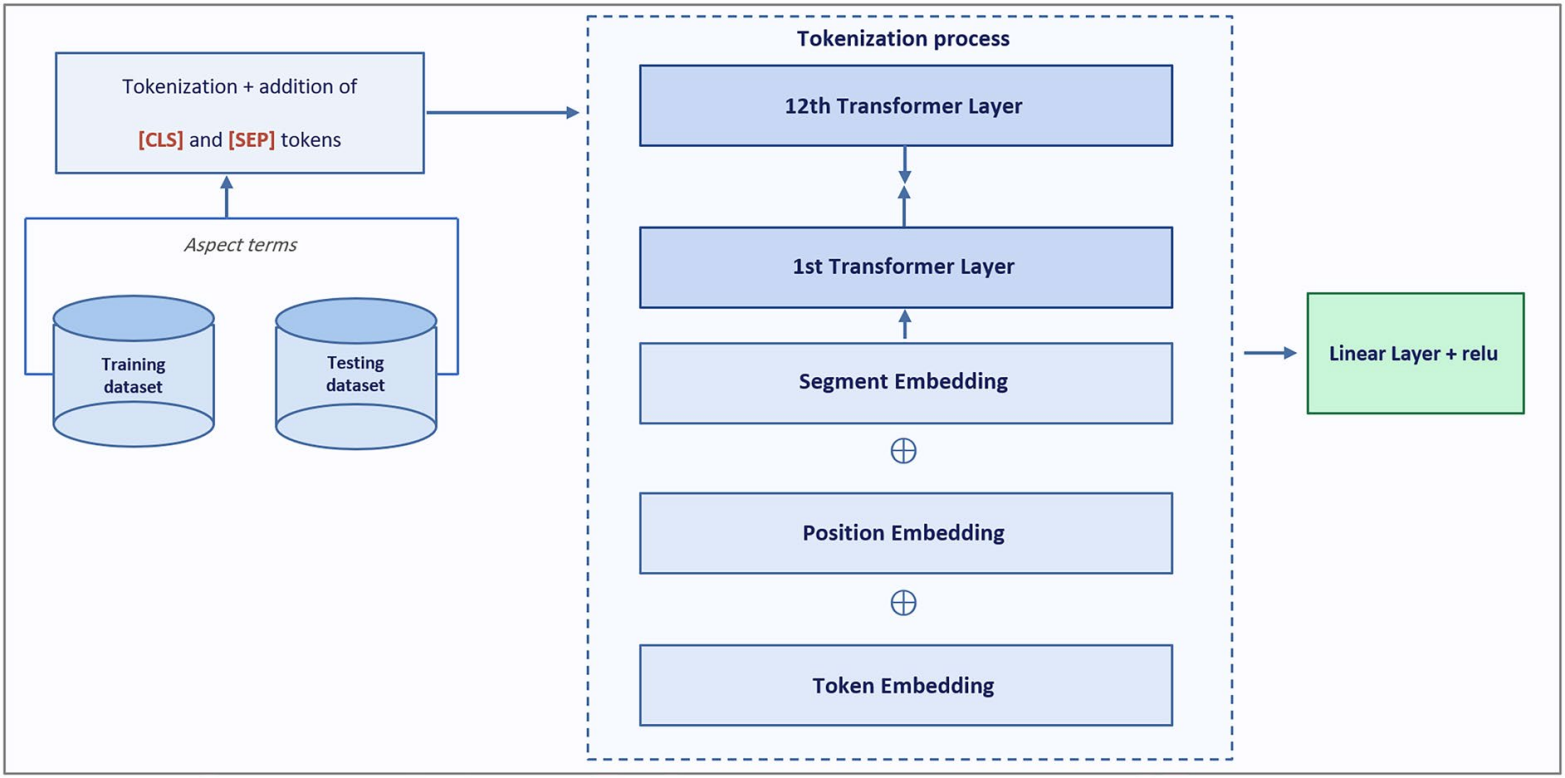
CAMeLBERT base components.

Texts are tokenized using CAMeLBERT’s WordPiece tokenizer. The special tokens [CLS] and [SEP] are added at the beginning and end of each sequence, respectively, following the BERT standard architecture^45,46^. Positional embeddings encode the order of tokens within the sequence, and segment embeddings are included for all tokens to indicate sentence membership, even when the sequence contains a single sentence. The sum of token, positional, and segment embeddings is passed through CAMeLBERT’s stacked transformer layers, producing contextualized token representations. The final hidden state corresponding to the [CLS] token (768-dimensional) is extracted as a fixed-length feature vector representing the entire sequence. These embeddings are then fed into a downstream feed-forward classifier, which applies a linear transformation followed by a ReLU activation function, as defined in Eq. 1 where the input value is denoted by $A$. Figure 2 illustrates the CAMeLBERT-based feature extractor architecture.

1 $$\begin{aligned} relu(A) = \max (0, A) \end{aligned}$$

#### DNN classifier

Text classification tasks demonstrate the advantage of NN for processing textual data^47^. A sequence of computational procedures is used by the numerous layers of linked neurons that make up the NN to convert input data into outputs^48^.

In this study, the CAMeLBERT model is employed to generate 768-dimensional contextualized embeddings from input text, which are then passed to the neural network for classification. The proposed classifier is a feed-forward neural network that takes these embeddings as input. The architecture consists of two fully connected layers with 256 and 64 units, respectively, each followed by ReLU activation and dropout (rate 0.2) for regularization. A final sigmoid layer outputs probabilities for binary classification, which are calculated as shown in Eq. 2. The network is trained using the Adam optimizer with binary cross-entropy loss, while class weights computed from the training set are applied to mitigate label imbalance.

2 $$\begin{aligned} \sigma (A) = \frac{1}{1 + e^{-A}} \end{aligned}$$

where $\sigma (A)$ denotes the output of the sigmoid function, which takes *A* as input, and *e* is the base of the natural logarithm, approximately 2.71828.

#### Model training setup

In this section, we describe the training configuration and systematic parameter tuning adopted for the hybrid CAMeLBERT-DNN system, including the definition of parameters and hyperparameters, to ensure clarity and reproducibility of the experimental process. In neural networks, parameters refer to the trainable weights and biases that the model learns during the training process, updated via backpropagation to minimize the loss function. Hyperparameters, on the other hand, are predefined settings, not learned from data, and are tuned experimentally for optimal performance, such as the learning rate, number of epochs, and batch size. Their selection is systematically justified in the following section.

**Hyperparameter Tuning:** The selection of the network architecture and the final hyperparameters was established via a structured **Grid Search methodology**^49^ conducted on the validation set, chosen to ensure an objective and reproducible, performance-driven selection process. This approach was adopted to systematically evaluate the impact of each hyperparameter on convergence behavior and generalization performance. Several configurations exhibited early signs of overfitting, which informed the exclusion of unstable settings. The final configuration was therefore selected based on its ability to minimize validation loss while maintaining stable generalization behavior. The hyperparameters explored included the learning rate $1 \times 10^{-5},\ 5 \times 10^{-5},\ 1 \times 10^{-4},\ 5 \times 10^{-4}$, batch size {16, 32, 64}, and variations in the DNN architecture, specifically the number of dense layer units {128, 256, 512} and corresponding dropout rates{0.1, 0.2, 0.3}. The final configuration (learning rate: $5 \times 10^{-4}$, batch size: 16, dense units: 256, dropout: 0.2) was validated as the selected setup, achieving efficient loss minimization without inducing training instability. The hyperparameter selection strategy was guided by standard practices in neural network tuning, where learning rates are explored on a logarithmic scale and batch sizes are chosen based on memory and generalization trade-offs^50^.

1. **Feature Extractor Selection:** We selected the CAMeLBERT-base MSA model as the foundational embedding layer. This choice was motivated by prior studies showing that CAMeLBERT-base MSA performs well on Modern Standard Arabic (MSA) tasks, compared to generic or dialectal models^6^. The selection process focused specifically on the MSA version of CAMeLBERT, as variants pre-trained on mixed dialects (e.g., CAMeLBERT-mix) or specific dialects (e.g., CAMeLBERT-da) are optimized for colloquial language tasks and were found to be suboptimal for our purely MSA-based corpus. Leveraging a model pre-trained exclusively on high-quality MSA corpus ensures that the rich morphological and syntactic features of the corpus are accurately captured, providing an optimized feature space for the downstream classification task.
2. **Imbalance Handling Justification:** As the dataset exhibits class imbalance, we opted for the algorithm-level solution of utilizing **Class Weighting**^43^. This technique, detailed in Section "Handling the imbalanced dataset problem using class weighting", modifies the Binary Crossentropy loss function to dynamically assign a higher penalty to errors made on minority-class samples. This method is preferred as it preserves the integrity of the original dataset distribution. The Class Weighting ($weight_i$) for each class *i* is determined based on the inverse frequency, as shown in Equation 3 where *N* is the total number of samples, *k* is the number of classes, and $n_i$ is the number of samples in class *i*.
  3 $$\begin{aligned} weight_i = \frac{N}{k \cdot n_i} \end{aligned}$$
3. **DNN Architecture and Activation:** The activation function used in the hidden layers is ReLU, while the final output layer employs the Sigmoid activation function for binary classification, which maps the network’s final output to a probability value [0, 1] facilitating the final decision on class membership. The ReLU activation was selected based on its computational efficiency and its established role in preventing vanishing gradients^51^. These choices (256 and 64 units, dropout rate 0.2) were made to balance the model’s expressive power and training efficiency while mitigating overfitting.
4. **Training Protocol:** The Max Sequence Length was fixed at 128 tokens based on the dataset’s token length distribution analysis, maximizing coverage while maintaining computational efficiency and minimizing the dilutive effect of zero-padding. The DNN training batch size was set to 16, providing a balance between memory efficiency and maintaining a stable gradient across updates. Different batch sizes were used for feature extraction and DNN training to account for their distinct computational and optimization characteristics. This distinction ensures both computational efficiency during feature extraction and stable gradient updates during DNN training. The model was trained using the Adam optimizer based on its established efficiency and status as the prevailing standard in deep learning literature for similar NLP tasks^52^ and was chosen with a learning rate of 0.0005, utilizing the Binary Crossentropy loss function, enhanced by Class Weighting (as fully described in Section "Handling the imbalanced dataset problem using class weighting") to mitigate label imbalance. The chosen learning rate was validated during the Grid Search process as the configuration that minimized the validation loss efficiently, without inducing training instability or requiring an excessive number of epochs. An initially higher maximum number of training epochs was employed in conjunction with Early Stopping based on the validation loss. Since convergence was consistently achieved before the tenth epoch, the number of epochs was fixed at 10 in the final training configuration. This strategy ensured efficient training while preventing overfitting and maintaining stable convergence behavior. The comprehensive set of experimental settings and hyperparameters used for the entire system are presented in Table 2 and Table 3.

**Table 2:** Experimental settings and parameters.

**Component**	**Parameter**	**Value/Setting**
Data Split	Test size	0.2 (20%)
	Training size	.8 (80%)
	Random state	44
Tokenizer	Model	CAMeL-Lab/bert-base-arabic-camelbert-msa
	Padding	max_length
	Truncation	True
Base Model	Model	CAMeLBERT-base MSA
	Embedding type	CLS token (last_hidden_state[:,0,:])
	Embedding size	$\sim$768
Evaluation	Metrics	Accuracy, Precision, Recall, F1-score, AUC, Confusion Matrix
Visualization	Curves	Training/Validation Loss & Accuracy
	Confusion Matrix Heatmap	cmap=”Blues”, annotated

**Table 3:** Hyperparameters.

**Component**	**Hyperparameter**	**Value/Setting**
Text Encoding	Max sequence length	128 tokens
Feature Extraction	Batch size (CAMeLBERT)	32
Neural Network	Dense layer 1 units	256, Activation = ReLU
	Dropout 1	0.2
	Dense layer 2 units	64, Activation = ReLU
	Dropout 2	0.2
	Output layer	1, Activation = sigmoid
Training	Optimizer	Adam (learning_rate=0.0005)
	Loss function	Binary crossentropy
	Epochs	10
	Batch size (DNN)	16
	Validation split	0.2 (20%)
	Class Weighting	Balanced

#### Hybrid classification algorithm (Pseudocode)

To ensure reproducibility and full transparency of the proposed hybrid approach, we present the complete implementation workflow, integrating all stages from data pre-processing and feature extraction to the final training and classification using the deep neural network. Algorithm 1 summarizes the step-by-step logic of the methodology.

**Algorithm 1:**
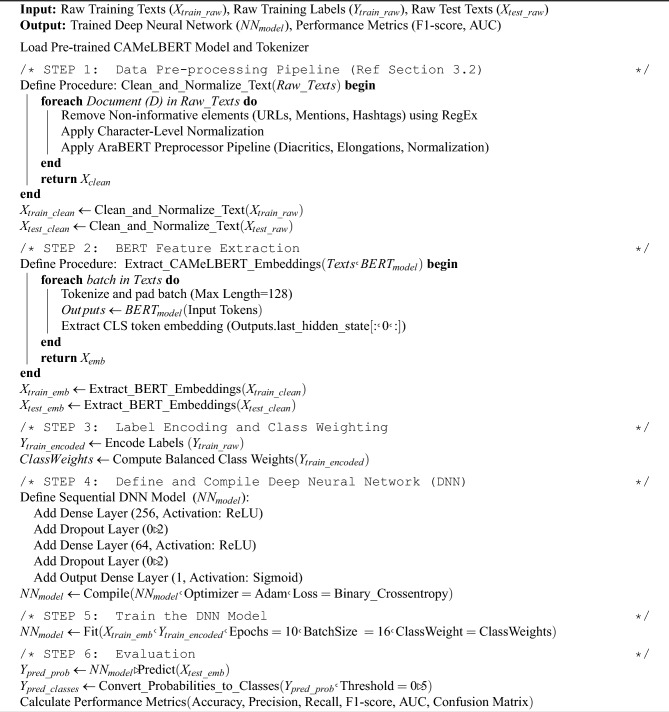
Training the hybrid CAMeLBERT-DNN classifier

### CAMeLBERT-ClassWeighting-NN evaluation metrics

The performance of the proposed hybrid CAMeLBERT-ClassWeighting-NN model is evaluated using standard classification metrics, including the confusion matrix, accuracy (ACCU), precision (PREC), recall (RECA), F1-score, and the area under the receiver operating characteristic curve (ROC-AUC). When estimating these metrics, we consider the number of true positives (|TP|), true negatives (|TN|), false positives (|FP|), and false negatives (|FN|).

A confusion matrix provides a detailed overview of the model’s performance on a test dataset, illustrating the counts of correctly and incorrectly classified instances^53^. This allows for the identification of specific types of misclassifications, offering insight into the strengths and weaknesses of the model^54^. Table 4 presents the generic structure of a confusion matrix. The actual counts used for evaluation are computed directly from the test set. The formulas for calculating the classification metrics are given in Equations (4)–(7). Each metric highlights a different aspect of model performance and is computed based on the confusion matrix derived from the test dataset. Higher values indicate better performance.

**Table 4:** Confusion Matrix Class Prediction Evaluation (1 = Real, 0 = Fake).

		Predicted Labels	
		**Class 0 (Fake)**	**Class 1 (Real)**
True Labels	**Class 0 (Fake)**	TN (True Negative)	FP (False Positive)
	**Class 1 (Real)**	FN (False Negative)	TP (True Positive)

4 $$\begin{aligned} \text {ACCU}= & \frac{|\text {TP}| + |\text {TN}|}{|\text {TP}| + |\text {FP}| + |\text {TN}| + |\text {FN}| } \end{aligned}$$

5 $$\begin{aligned} \text {PREC}= & \frac{|\text {TP}|}{|\text {TP}| + |\text {FP}| } \end{aligned}$$

6 $$\begin{aligned} \text {RECA}= & \frac{|\text {TP}|}{|\text {TP}| + |\text {FN}| }\end{aligned}$$

7 $$\begin{aligned} \text {F1-score}= & 2 * \frac{\text {Precision} * \text {Recall}}{\text {Precision} + \text {Recall} } \end{aligned}$$

While the previous metrics quantify performance at a fixed threshold, the ROC-AUC evaluates model performance across varying classification thresholds, providing insight into the model’s discriminative ability. In addition, the ROC curve is used to evaluate the trade-off between the true positive rate (recall) and the false positive rate across different classification thresholds. AUC provides a summary measure of the model’s discriminative ability.

### Model interpretability

To improve the transparency and explainability of the proposed CAMeLBERT-based neural network framework, model interpretability techniques were employed. This is particularly crucial in fake news detection, where understanding the linguistic indicators of deception is as important as the classification accuracy itself. These techniques aim to provide insights into how different input features, represented as contextual embeddings, influence the model’s predictions. In this study, two complementary approaches were utilized: SHAP for global interpretability, which identifies overall patterns and influential embedding dimensions across the dataset, and LIME for local interpretability, which explains individual predictions by approximating the model’s behavior around specific instances. This dual approach enables a comprehensive understanding of the model’s decision-making process at both the dataset and instance levels.

#### SHAP (global interpretability)

To enhance the transparency and interpretability of the proposed classification framework, SHAP (SHapley Additive exPlanations) was employed to analyze the contribution of input features to the model’s predictions. Based on cooperative game theory, SHAP provides a mathematically grounded approach to attribute the model’s output to each feature’s contribution. Since the classifier relies on fixed contextual embeddings extracted from CAMeLBERT, interpretability is performed at the embedding level rather than at the raw token level.

A neural network classifier was explained using SHAP DeepExplainer, which is specifically optimized for deep learning architectures. A representative background set was constructed by randomly sampling up to 100 embedding vectors from the training data to estimate the expected model output. For global interpretation, SHAP values were computed for a randomly selected subset of up to 500 samples from the test set. Global feature importance was assessed using SHAP summary plots, including bar plots for mean absolute SHAP values and dot plots for the distribution and direction of contributions across samples.

#### LIME (local interpretability)

To provide instance-level explanations, LIME (Local Interpretable Model-agnostic Explanations) was applied to selected test instances. LIME approximates the classifier locally with a simpler interpretable model to identify which embedding dimensions contributed most significantly to individual predictions. By perturbing the input embeddings and observing the changes in the model’s output, LIME provides a ”human-friendly” explanation for why a specific Arabic article was flagged as fake. This local analysis complements the global insights obtained from SHAP, enabling a more detailed understanding of the model’s decision-making process for specific samples.

## Experimental results

This section reports the experimental evaluation of the proposed hybrid CAMeLBERT-ClassWeighting-NN model and presents a comparative analysis against baseline methods and Transformer-based approaches. The primary objective is to assess the effectiveness of contextual CAMeLBERT embeddings and the impact of class imbalance handling on Arabic fake news detection.

To ensure the reliability of the comparative analysis, all experiments were conducted under standardized conditions using a Tesla T4 GPU with 12 GB memory. The implementation was carried out using Python 3.10.12 and PyTorch 2.2.1. The CAMeLBERT-ClassWeighting-NN model was trained using the AdamW optimizer with a maximum sequence length of 128 tokens, a batch size of 16, and for 10 epochs.

### Baseline models performance

To evaluate the effectiveness of the proposed framework, we first compared the performance of the neural network (NN) baseline against several traditional machine learning algorithms, namely K-Nearest Neighbors (KNN), Multinomial Naive Bayes (MNB), and Decision Tree (DT). For all baseline models, the TF-IDF (Term Frequency-Inverse Document Frequency) method was applied to convert the textual data into numerical features. Unigrams and bigrams were extracted with a maximum of 2000 features, generating fixed-length vectors that effectively capture term importance across the dataset.

Table 5 summarizes the performance metrics of the baseline models, including accuracy (ACCU), precision (PREC), recall (RECA), F1-score, and ROC-AUC. Among these, the NN consistently outperformed the classical algorithms, achieving the highest overall accuracy (90.97%) and F1-score (92.20%). The Decision Tree achieved competitive results (ACCU = 88.03%, F1-score = 89.73%), while KNN and MNB showed lower performance across most metrics.

The superior performance of the NN over traditional classifiers can be attributed to its ability to model high-dimensional feature spaces and capture non-linear dependencies between TF-IDF features that linear models like MNB or distance-based models like KNN might overlook. This strong balance between precision and recall demonstrates the neural network’s ability to generalize effectively, even in the presence of class imbalance, and underscores the advantages of non-linear transformations in capturing complex patterns within Arabic news text.

**Fig. 3:**
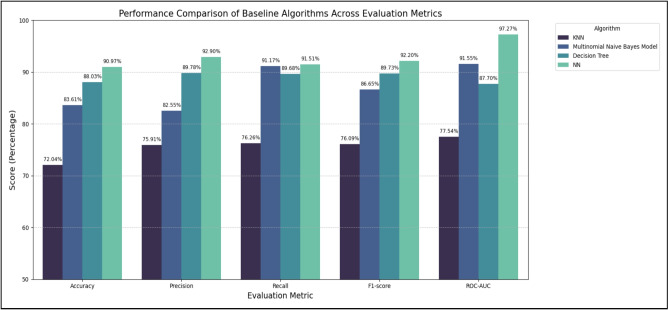
Performance metrics for the baseline models.

The high ROC-AUC (97.27%) further suggests that the NN has a strong discriminatory power, minimizing the trade-off between false positives and false negatives. Consequently, the NN baseline served as a reference for evaluating the added value of contextual embeddings from Transformer-based models such as AraBERT and CAMeLBERT, and was ultimately selected as the primary baseline for subsequent experiments in the proposed framework. For clarity, the NN baseline in Table 5 corresponds to the TF-IDF + NN entry in Table 6, providing a direct comparison between traditional feature-based methods and Transformer-based embeddings. The graphical representation of these results is presented in Fig. 3.

**Table 5:** Performance evaluation metrics for the baseline algorithms.

Algorithm	Evaluation metrics				
	ACCU	PREC	RECA	F1-score	ROC-AUC
KNN	72.04%	75.91%	76.26%	76.09%	77.54%
Multinomial Naive Bayes Model	83.61%	82.55%	91.17%	86.65%	91.55%
Decision Tree	88.03%	89.78%	89.68%	89.73%	87.70%
NN	90.97%	92.90%	91.51%	92.20%	97.27%

### Transformer embeddings performance

To assess the impact of contextual embeddings on Arabic fake news detection, several Transformer-based models were evaluated in combination with the neural network classifier. The models considered include AraELECTRA, MARBERTv2, AraBERT, and CAMeLBERT (MSA). AraELECTRA relies on a token discrimination objective (replaced token detection) rather than masked language modeling, providing competitive recall but occasionally exhibiting lower precision. MARBERTv2 was pre-trained on a large combined corpus including Modern Standard Arabic and other sources such as AraNews, enabling it to handle longer contexts effectively. AraBERT is specifically designed for Arabic text and demonstrates strong performance on MSA, particularly in identifying fake news instances. CAMeLBERT (MSA) is pre-trained on an extensive corpus of Modern Standard Arabic, capturing nuanced contextual representations, which explains its superior performance in this task.

Table 6 presents the performance metrics, including accuracy (ACCU), precision (PREC), recall (RECA), F1-score, and ROC-AUC. Among these models, CAMeLBERT achieves the strongest overall performance with a robust balance between metrics, with an accuracy of 93.98%, F1-score of 95.05%, and ROC-AUC of 98.65%. Notably, CAMeLBERT exhibits an exceptional Recall of 99.08%, significantly outperforming the TF-IDF + NN baseline by 7.57% and surpassing AraBERT by 1.37%. While AraBERT also demonstrates strong results, particularly in recall (97.71%), it achieves a lower F1-score (94.61%) compared to CAMeLBERT. In contrast, AraELECTRA, while showing competitive recall (97.25%), exhibits lower precision, resulting in a slightly reduced F1-score.

The superiority of CAMeLBERT + NN can be attributed to the model’s specialized pre-training on high-quality MSA corpora, which allows it to generate more ”discriminative” embeddings for formal news content. While some models show higher individual metrics in certain runs, the CAMeLBERT + NN configuration achieved the highest F1-score. This provides the most stable and balanced performance for real-world deployment, where both false alarms and missed detections must be minimized. These results clearly highlight the advantage of using CAMeLBERT embeddings over both the traditional TF-IDF baseline and other Transformer models, emphasizing its ability to capture nuanced contextual information in Arabic text. The high F1-score (95.05%) and recall (99.08%) achieved by CAMeLBERT are critical, as fake news in Arabic often employs subtle linguistic manipulations, semantic distortions, and stylistic nuances that require models with sophisticated contextual understanding. This result validates CAMeLBERT’s specific training on Modern Standard Arabic for robustness against these linguistic complexities and proves its superior capability in modeling the subtle semantic shifts often present in deceptive Arabic content. Furthermore, these findings have significant practical implications, suggesting that while AraBERT offers high sensitivity, CAMeLBERT provides a more stable and reliable deployment solution. The superior ROC-AUC (98.65%) indicates that the model has developed a clear boundary between deceptive and legitimate content, minimizing the operational cost of manual fact-checking. For news agencies, adopting CAMeLBERT translates to a more autonomous system that filters misinformation with high confidence without overwhelming human moderators with false alarms.

**Fig. 4:**
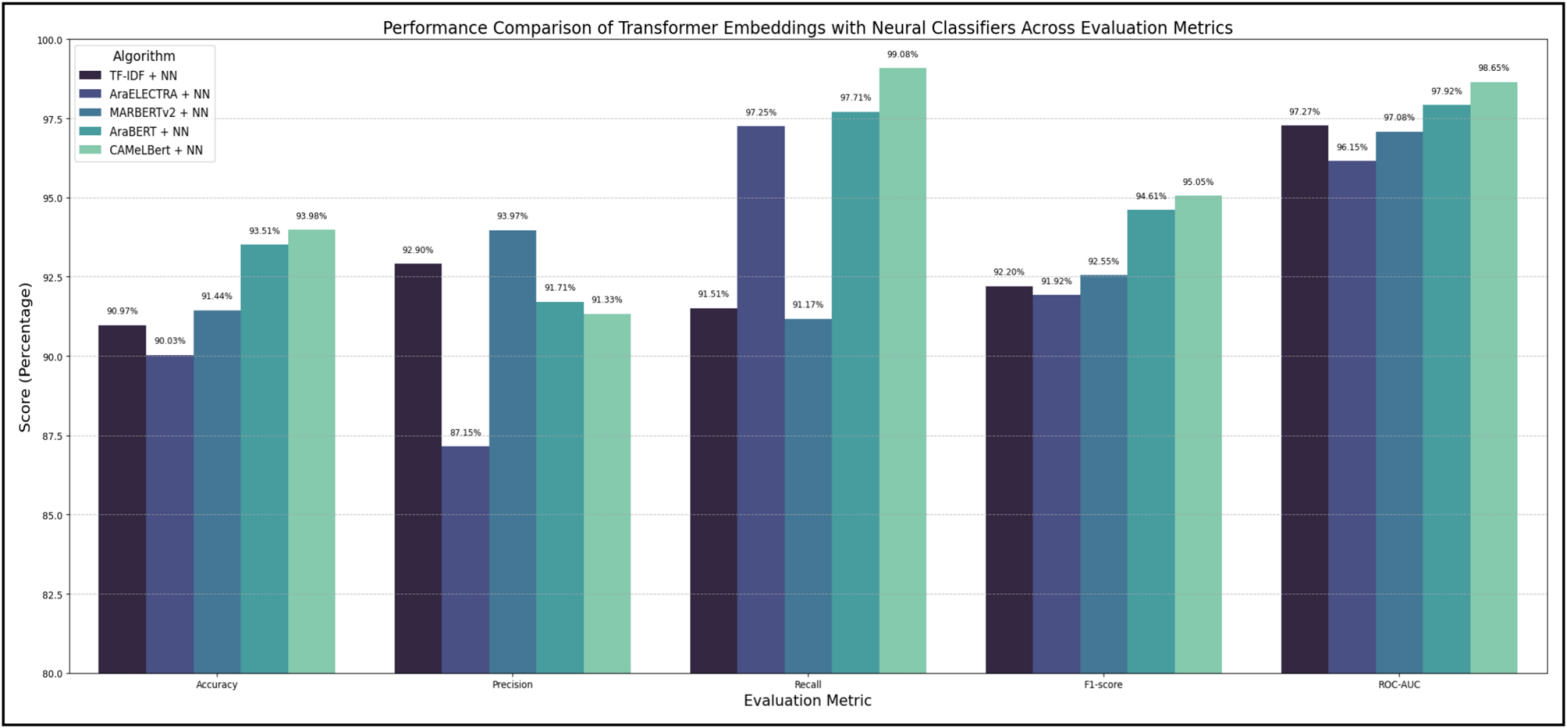
Performance metrics of transformer embeddings with neural classifier.

Figure 4 illustrates the comparative performance of all Transformer embeddings combined with the neural network classifier, providing a visual representation of the improvements achieved.

**Table 6:** Comparison of transformer embeddings with neural classifiers for Arabic fake news detection.

Algorithm	Evaluation metrics				
	ACCU	PREC	RECA	F1-score	ROC-AUC
TF-IDF + NN	90.97%	92.90%	91.51%	92.20%	97.27%
AraELECTRA + NN	90.03%	87.15%	97.25%	91.92%	96.15%
MARBERTv2 + NN	91.44%	93.97%	91.17%	92.55%	97.08%
AraBERT + NN	93.51%	91.71%	97.71%	94.61%	97.92%
CAMeLBERT + NN	93.98%	91.33%	99.08%	95.05%	98.65%

### Top-performing models with resampling and class imbalance techniques

To further enhance the detection performance of Arabic fake news, several resampling strategies and class imbalance handling techniques were applied to the top-performing CAMeLBERT-based neural network model. The approaches considered include oversampling, undersampling, SMOTE (Synthetic Minority Over-sampling Technique), and Class Weighting. Table 7 presents the performance metrics of these setups.

Among these combinations, CAMeLBERT + Class Weighting + NN achieves the most robust and stable overall performance, reaching an accuracy of 95.52%, F1-score of 96.19%, and a precision of 95.48%. While the SMOTE-based model also demonstrates strong results (F1-score = 95.71%), Class Weighting proves superior by maintaining the original distribution of the data while penalizing misclassifications of the minority (fake news) class. This avoids the potential risk of overfitting associated with oversampling and the information loss inherent in undersampling, which explains the latter’s lower accuracy (92.51%) due to the removal of potentially discriminative majority-class samples that are essential for the transformer to learn the nuances of legitimate Arabic news.

Notably, the introduction of Class Weighting significantly improved the model’s Precision (from 91.33% to 95.48%) compared to the baseline CAMeLBERT model. This suggests that by adjusting the loss function, the model became more ”conservative” and precise in its predictions, effectively reducing the false positive rate (classifying fake news as real). Although there was a slight, expected decrease in Recall (from 99.08% to 96.90%), the substantial gain in precision led to a higher and more balanced F1-score. This trade-off is strategically advantageous for deployment, as it significantly reduces ”false alarms” without compromising the system’s sensitivity to deceptive content. These results highlight that addressing class imbalance at the algorithmic level is more effective for Arabic fake news detection than traditional data-level resampling.

**Fig. 5:**
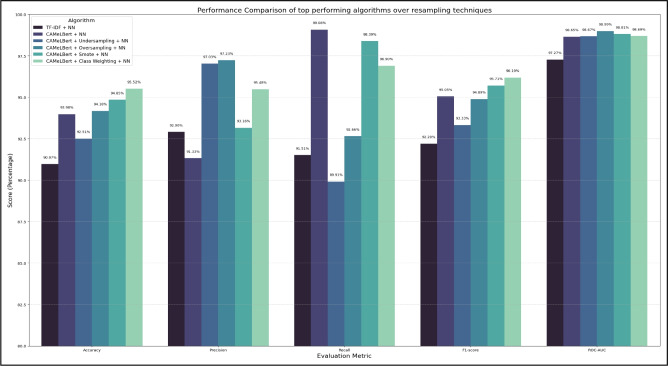
Performance metrics for top-performing models under resampling techniques.

Unlike SMOTE, which generates synthetic Arabic embeddings that might not correspond to semantically coherent text, Class Weighting operates on the actual, verified contextual representations. This makes it the most suitable strategy for real-world deployment, where maintaining data integrity is crucial. Furthermore, the consistent ROC-AUC values across all resampling configurations (exceeding 98.6%) confirm that the CAMeLBERT-based architecture maintains high discriminative power regardless of the sampling strategy. However, the superior F1-score of the Class Weighting approach reinforces its role in achieving the optimal trade-off between identifying fake news and preserving legitimate content. Figure 5 provides a comparative analysis of the top-performing models under different resampling and class imbalance techniques.

**Table 7:** Performance evaluation metrics for the top-performing models.

Algorithm	Evaluation metrics				
	ACCU	PREC	RECA	F1-score	ROC-AUC
TF-IDF + NN	90.97%	92.90%	91.51%	92.20%	97.27%
CAMeLBERT + NN	93.98%	91.33%	99.08%	95.05%	98.65%
CAMeLBERT + Undersampling + NN	92.51%	97.03%	89.91%	93.33%	98.67%
CAMeLBERT + Oversampling + NN	94.18%	97.23%	92.66%	94.89%	98.99%
CAMeLBERT + SMOTE + NN	94.85%	93.16%	98.39%	95.71%	98.81%
CAMeLBERT + Class Weighting + NN	95.52%	95.48%	96.90%	96.19%	98.69%

### Training and stability analysis of baseline model (TF-IDF + NN)

**Fig. 6:**
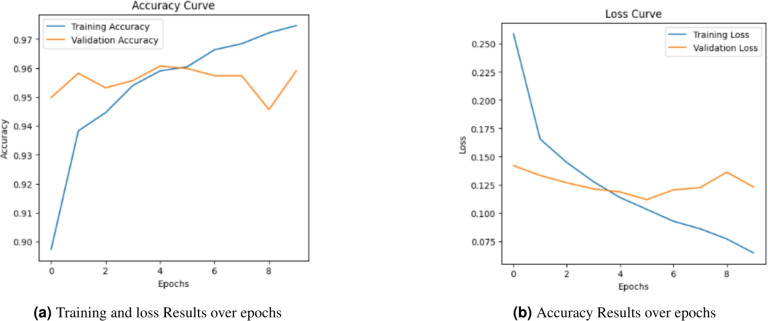
Performance evaluation of training loss and accuracy for TF-IDF + NN model.

To gain deeper insights into the training dynamics and generalization capability of the TF-IDF + NN baseline model, the Loss and accuracy curves over 10 epochs were analyzed in Fig. 6.

The analysis of the Accuracy and Loss curves reveals a clear pattern of overfitting and high variance. While the training accuracy rapidly approaches 1.00 and Training Loss drops near zero, a critical divergence is observed in the validation metrics. As shown in Fig. 6, the validation loss begins to increase significantly after the first epoch,reaching its peak at the final epoch. This growing gap is a classic indicator that the model has reached its generalization capacity and has transitioned into memorizing statistical noise and specific keyword frequencies within the training set, rather than extracting robust semantic patterns. This limitation is fundamentally inherent to the sparse nature of TF-IDF representations, which fail to capture the complex morphological dependencies and long-range contextual cues essential for the Arabic language.

Practical Implication of Overfitting: The observed overfitting suggests that while the model performs well on familiar data, its reliability in a real-world environment–where Arabic news content is diverse, evolving, and often characterized by subtle linguistic manipulations–would be severely compromised. The instability shown in the loss curve warns of poor production performance, as the model lacks the ”semantic depth” to generalize to novel deceptive styles.

**Fig. 7:**
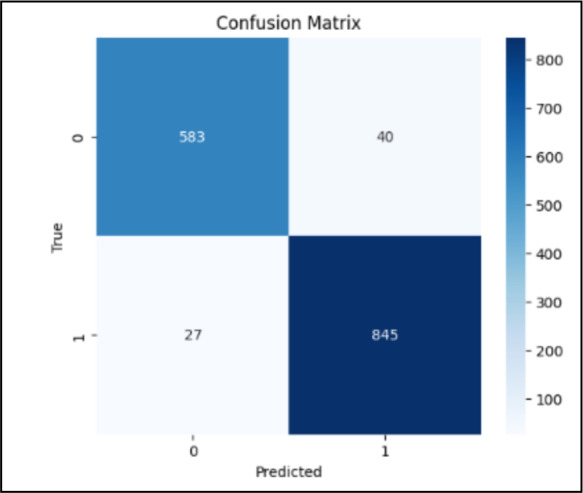
Confusion matrix for the TFIDF + NN baseline model.

The confusion matrix in Fig. 7 provides a granular view of these classification errors. Out of the test set, the model correctly identified 562 fake articles (True Negatives) and 798 real articles (True Positives). However, the presence of 61 False Positives (fake news incorrectly flagged as real) represents a significant risk to information integrity, while 74 False Negatives indicate that legitimate content is being erroneously penalized. This error rate, coupled with the instability observed in the training curves, underscores the ”semantic gap” present in frequency-based approaches. These findings reinforce the necessity of transitioning to a Transformer-based architecture to provide the nuanced contextual understanding required for stable and reliable Arabic fake news detection.

### Training dynamics and comparative analysis of contextual embeddings

The transition from static frequency-based features to contextual transformer embeddings marks a significant improvement in capturing the semantic nuances of the Arabic language. This section analyzes the performance of two state-of-the-art models, AraBERT and CAMeLBERT, both integrated with a neural network classifier to overcome the limitations of traditional models.

#### CAMeLBERT dynamics and stability

**Fig. 8:**
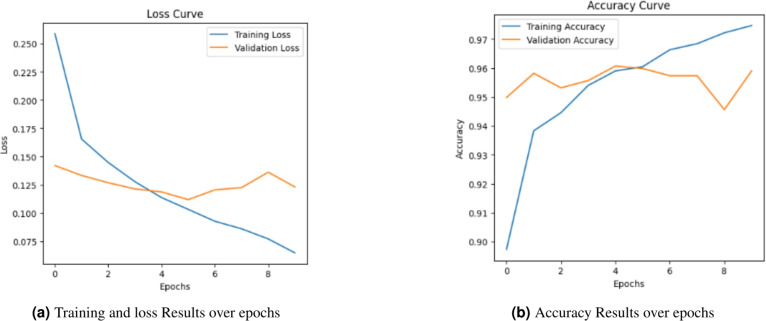
Performance evaluation of training loss and accuracy for the CAMeLBERT + NN model.

As illustrated in Fig. 8, the CAMeLBERT + NN model demonstrates superior stability and learning efficiency compared to the TF-IDF baseline. The validation accuracy significantly improves, reaching 93.98%, while the training and validation loss curves show a much more synchronized convergence in the initial epochs. Unlike the baseline model, which suffered from immediate and drastic divergence, CAMeLBERT maintains a tighter gap between training and validation metrics. Although a slight uptick in validation loss is observed after epoch 8, the model’s overall generalization remains robust. This indicates that the transformer architecture effectively leverages its self-attention mechanism to capture deep semantic relationships and morphological dependencies, rather than merely memorizing vocabulary patterns.

#### Comparative error analysis (AraBERT vs. CAMeLBERT)

To justify the selection of the optimal backbone for our hybrid system, a comparative error analysis was conducted using the confusion matrices of both transformer-based models. While the AraBERT + NN model achieved solid performance, it exhibited a higher rate of misclassifications in the Real class, recording 20 False Negatives (actual real news incorrectly classified as fake). In contrast, CAMeLBERT + NN demonstrated a superior ability to identify legitimate content, drastically reducing False Negatives to only 8 cases. Although CAMeLBERT recorded 82 False Positives (actual fake news misclassified as real), slightly higher than AraBERT’s 77, its overall stability and superior ROC-AUC (98.65% vs 97.92%) establish it as the more reliable foundation. As summarized in Table 8, CAMeLBERT outperforms AraBERT across all key metrics, particularly in F1-Score (95.05%), which is critical given the moderate class imbalance of the dataset.

**Table 8:** Comparative error metrics: AraBERT vs. CAMeLBERT.

Model	FN	FP	Accuracy	F1-Score	ROC-AUC
AraBERT + NN	20	77	93.51%	94.61%	97.92%
CAMeLBERT + NN	**8**	82	**93.98%**	**95.05%**	**98.65%**

#### Practical implication of errors and optimization needs

**Fig. 9:**
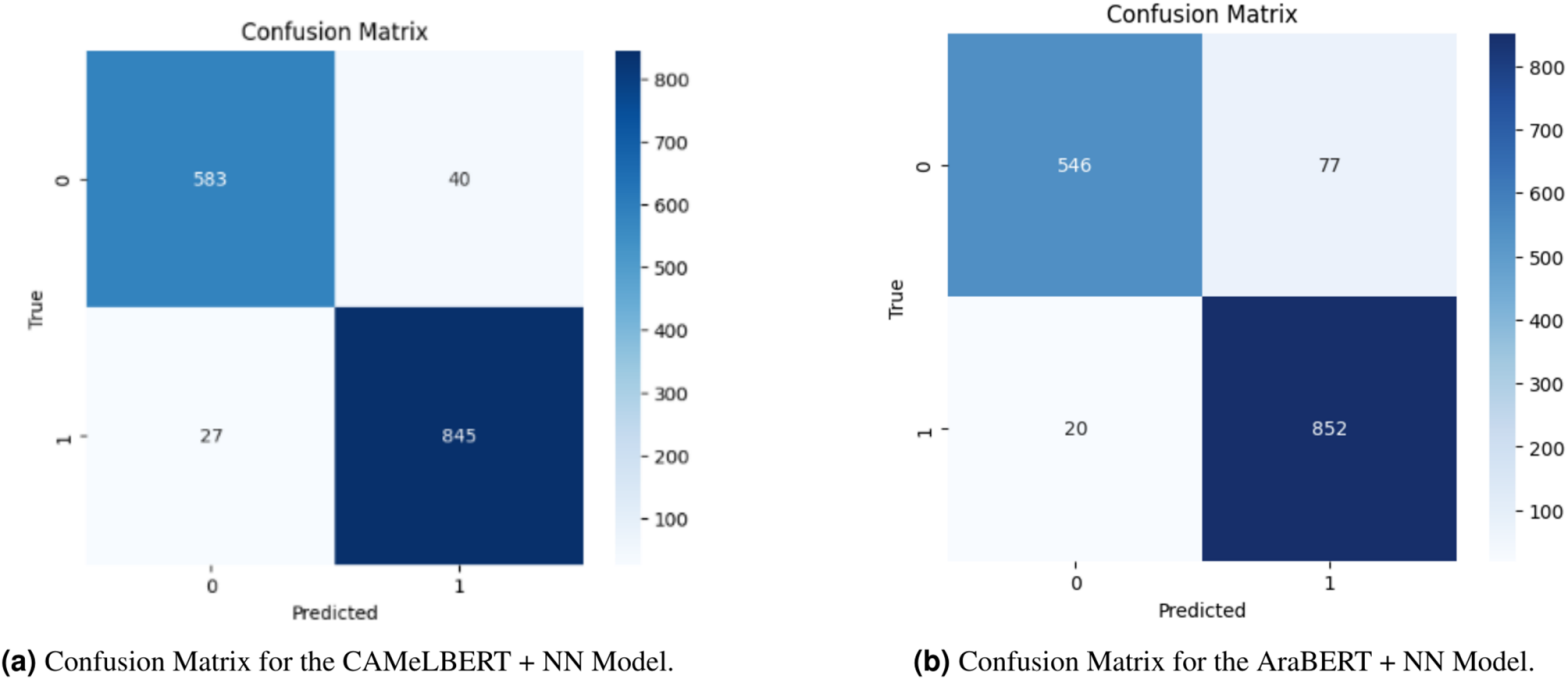
Comparative Confusion Matrices of CAMeLBERT+NN and AraBERT+NN Models.

Despite the high performance of both models, a common challenge remains: a subtle bias toward the majority class (real news). As illustrated in the Confusion Matrices (Fig. 9), the CAMeLBERT model recorded 82 False Positives–malicious articles that bypassed detection–representing a critical ”Type I Error” (False Acceptances). This poses a significant real-world risk, as undetected misinformation can spread without intervention. While CAMeLBERT successfully shifted the error distribution to protect legitimate content–reducing False Negatives to only 8–it retained a residual imbalance that prioritizes overall accuracy over precision toward the minority (fake) class. This necessitates the final refinement phase in Section "Final optimization and performance of the optimal model (CAMeLBERT + Class Weighting + NN)", where Class Weighting is applied to penalize the misclassification of fake news, aiming for a more security-oriented and balanced performance.

### Final optimization and performance of the optimal model (CAMeLBERT + class weighting + NN)

The final stage of optimization addressed the class imbalance identified in the baseline CAMeLBERT model (FP = 82). To achieve a more balanced performance, Class Weighting was implemented as a cost-sensitive learning strategy. This approach allowed the model to give appropriate importance to the minority ”Fake” class during the training process, leading to the development of a more discriminative and secure decision boundary.

#### Analysis of training dynamics and stability

**Fig. 10:**
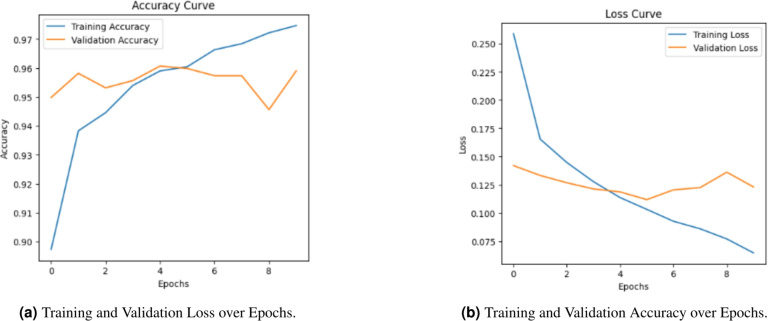
Performance evaluation of training loss and accuracy for the CAMeLBERT + Class Weighting + NN optimal model.

The Accuracy and Loss curves (Fig. 10) confirm the continuous stability of the optimized model. Unlike traditional resampling techniques which can often introduce noise or training instability, the validation loss remains low and consistent throughout the training process. This stability, culminating in a peak validation accuracy of 95.52%, demonstrates that Class Weighting effectively recalibrates the model’s sensitivity without compromising its generalization capability or reintroducing fluctuations.

#### Final performance evaluation and error balance

**Fig. 11:**
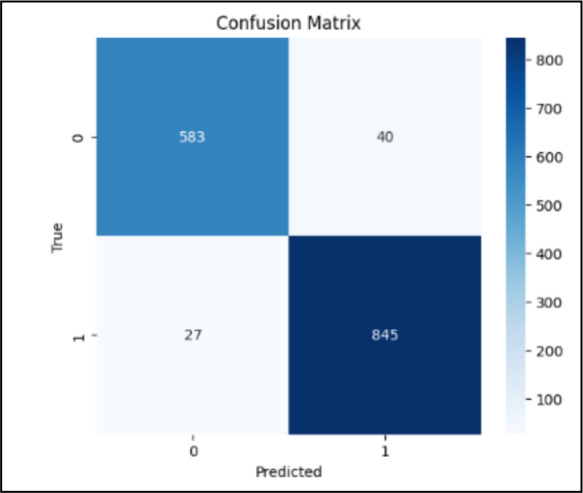
Confusion Matrix and Final Performance Metrics for the Optimal CAMeLBERT + Class Weighting + NN Model.

The Confusion Matrix (Fig. 11) and the metrics in Table 7 highlight the successful re-establishment of the model’s predictive balance through the following key observations:

- **False Positives (FP) Reduction:** The number of critical errors (fake news misclassified as real) dropped sharply from 82 to 40, marking a 51.2% improvement in system security. This reduction is vital for mitigating the real-world risk of undetected misinformation.
- **Optimal Error Trade-off:** While the False Negatives (FN) adjusted from 8 to 27, this represents a necessary and scientifically justified trade-off in imbalanced learning. It signifies the transition from a model biased toward the majority class to a balanced system that prioritizes ”precision-recall” equilibrium.
- **Overall High-Performance Metrics:** As summarized in Table 7, the final configuration achieved the highest overall accuracy of 95.52% and the leading F1-score of 96.19% among all evaluated models. Although the ROC-AUC of 98.69% is marginally lower than that of the oversampling method (98.99%), this slight difference is strategically outweighed by the model’s superior training stability and higher F1-score. Since the F1-score provides a more robust reflection of predictive power in imbalanced datasets, these results confirm that Class Weighting establishes the most reliable and well-balanced decision boundary for detecting deceptive content.

#### Analysis of error evolution across models

Table 9 provides a comprehensive overview of the performance evolution throughout the study. The comparative analysis documents the transition from frequency-based heuristics to a cost-sensitive, contextual deep learning framework through three critical phases of the research:

1. **Baseline Performance:** The TF-IDF + NN model established a preliminary performance level, where FN (74) and FP (61) errors were proportionally high relative to the total samples. This confirmed that static, frequency-based features lack the semantic depth required to distinguish between legitimate reporting and sophisticated Arabic misinformation.
2. **Feature Dominance and Bias:** The shift to CAMeLBERT + NN significantly improved the overall F1-score to 95.05%. However, it exposed a distinct majority-class bias; while the transformer architecture proved highly sensitive in identifying legitimate news (FN=8), the sharp rise in False Positives (FP=82) indicated a decision boundary skewed toward the ”Real” class, leading to the misclassification of deceptive content.
3. **Optimal Balance and Performance:** The final CAMeLBERT + Class Weighting + NN configuration successfully rectified this imbalance. By achieving a superior error equilibrium (FN = 27, FP = 40), the model halved the critical Type I errors (False Positives) while maintaining the highest accuracy (95.52%) and F1-score (96.19%). This demonstrates that the deep contextual extraction power of CAMeLBERT was effectively leveraged, while the decision boundary was optimally calibrated by the Class Weighting strategy to ensure a more security-centric performance.

**Table 9:** Confusion matrix and classification performance of evaluated models.

Model Configuration	Predicted Fake News		Predicted Real News		Accuracy	F1-score
	True Negative	False Negative	False Positive	True Positive		
**TF-IDF + NN (Baseline)**	562	74	61	798	90.97%	92.20%
**CAMeLBERT + NN**	541	8	82	864	93.98%	95.05%
**CAMeLBERT + CW + NN**	**583**	**27**	**40**	**845**	**95.52%**	**96.19%**

### Model interpretability: bridging contextual embeddings and decision logic

To meet transparency requirements and provide a principled justification for the proposed model’s predictions, we developed a dedicated interpretability pipeline that elucidates how high-dimensional latent representations produced by the CAMeLBERT encoder are transformed into final classification probabilities by the downstream neural network classifier. This analysis bridges the gap between contextual embedding spaces and model decision logic at both global and local levels.

#### Global feature attribution (SHAP analysis)

Global interpretability was conducted using SHAP DeepExplainer, which is well-suited for deep neural architectures operating on dense continuous representations. Rather than attributing importance to surface-level linguistic tokens, this analysis focuses on the 768-dimensional latent contextual embedding space generated by CAMeLBERT.

**Fig. 12:**
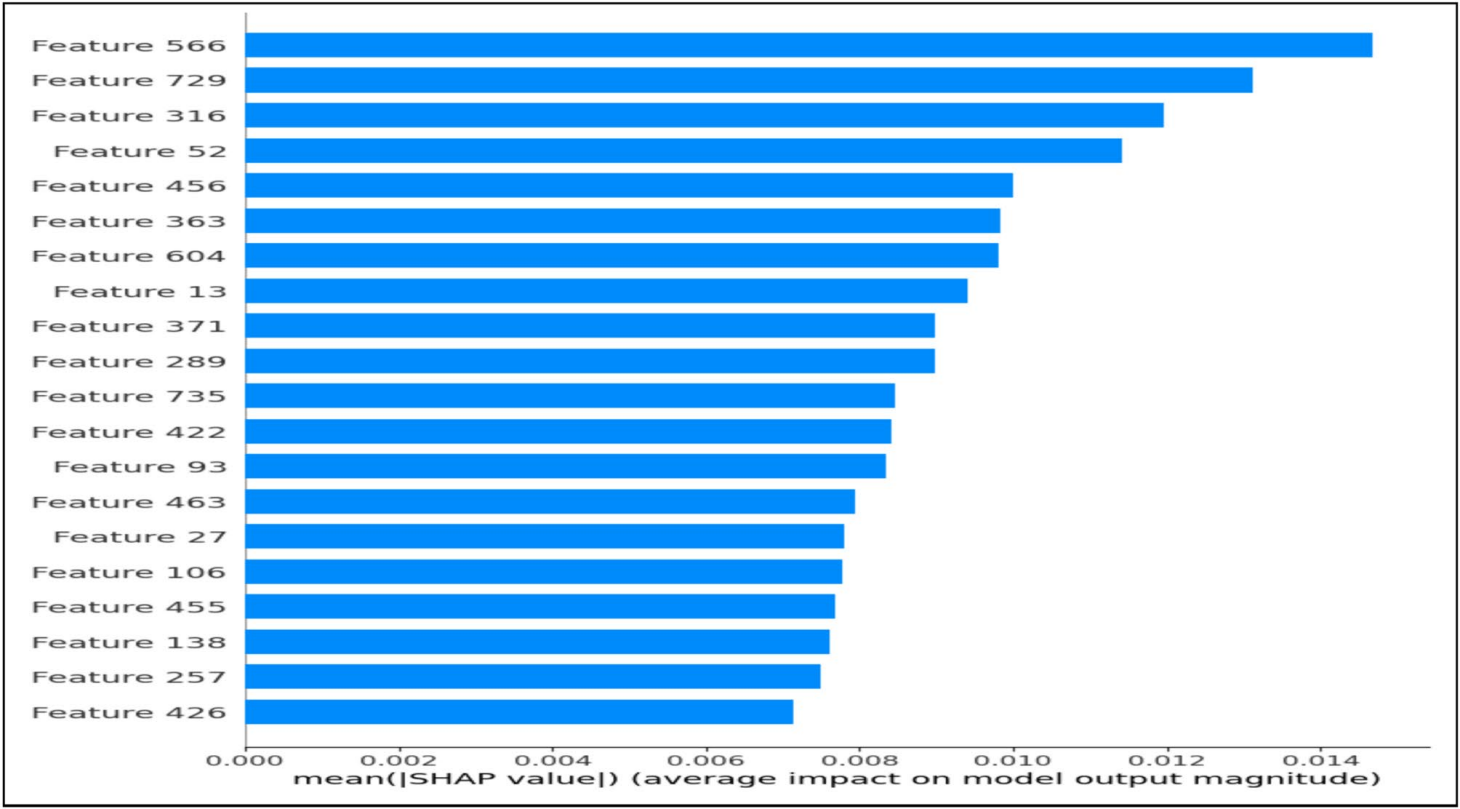
SHAP global feature importance bar plot. Top 20 embedding dimensions.

**Fig. 13:**
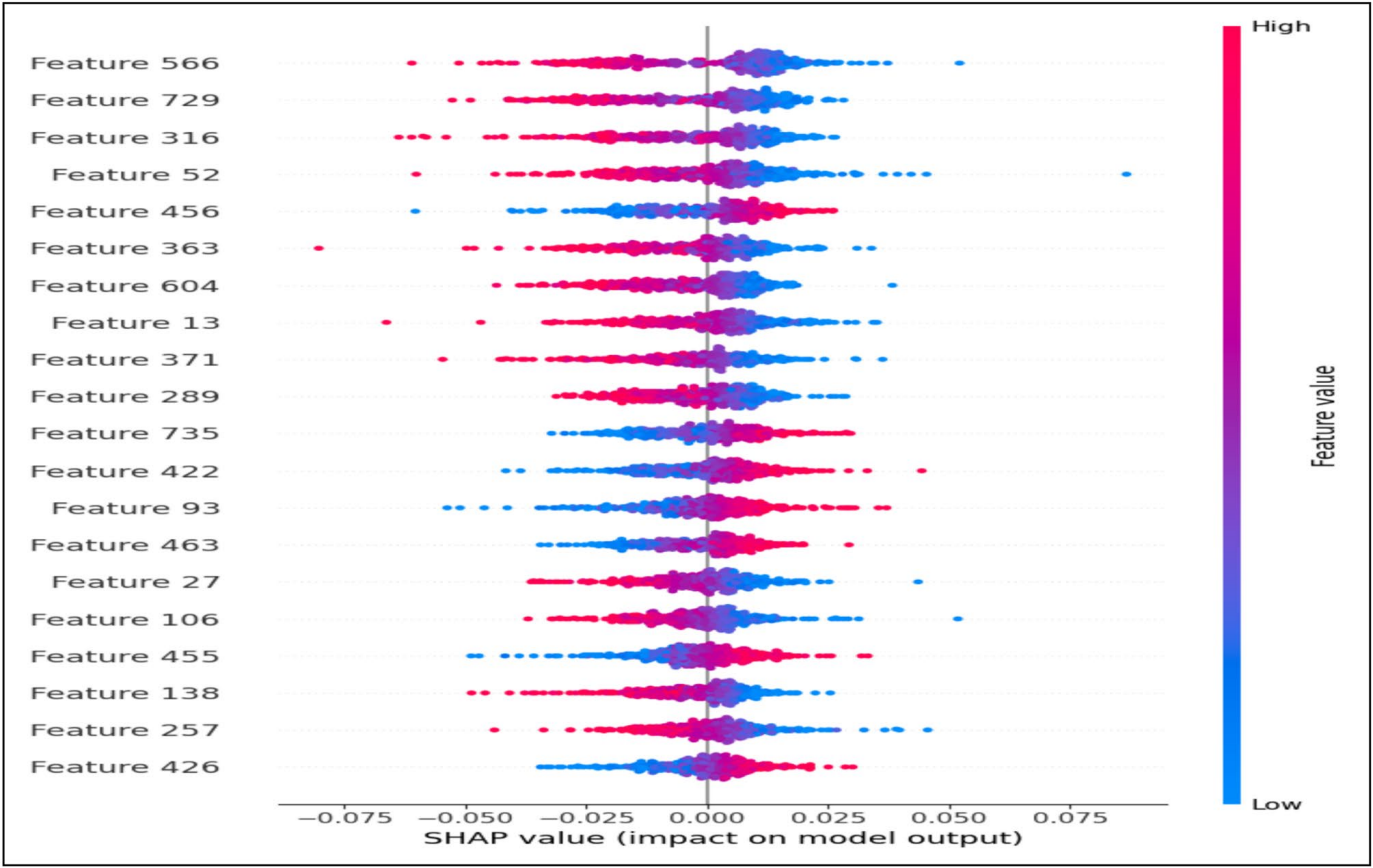
SHAP Summary Dot Plot, Distribution of SHAP values across samples and the direction of influence.

- **Methodology:** The explainer was initialized with a representative background distribution consisting of 100 embedding samples randomly drawn from the training set ($\textbf{X}_{train\_emb}$), establishing a stable reference baseline. Shapley values were subsequently computed for 500 unseen test instances to ensure statistical robustness and reduce sampling variance.
- **Findings:** As illustrated in Fig. 12, the SHAP summary plot highlights specific latent dimensions (e.g., Feature 566 and Feature 729) as having consistently high influence on the model’s predictions. These dimensions do not correspond to explicit or human-interpretable linguistic features; instead, they capture abstract semantic interactions learned during CAMeLBERT’s pre-training and fine-tuning stages. The non-sparse distribution of SHAP values across dimensions confirms that the classifier exploits the full contextual richness of the embedding space, enabling the modeling of complex deceptive patterns that cannot be adequately represented using traditional sparse features such as TF–IDF.
- **Decision Boundary Calibration:** The beeswarm visualization (Fig. 13) further suggests that the application of class weighting (CW) influenced the relative contribution of embedding dimensions associated with the minority (fake news) class. This redistribution of feature attributions indicates a calibrated decision boundary that emphasizes embedding signals correlated with misleading content, contributing to a measurable reduction in false positive predictions. While this effect reflects statistical associations rather than strict causality, it provides empirical evidence that CW alters how semantic information is leveraged during classification.

#### Local linguistic evidence (LIME implementation)

To complement the global SHAP analysis, local interpretability was achieved using LIME to explain individual predictions at the textual level. Since LIME natively operates on raw text inputs, whereas the proposed model consumes dense embeddings, a custom prediction wrapper (predict_proba_texts) was implemented. This wrapper dynamically converts LIME-generated text perturbations into CAMeLBERT embeddings in real-time batches before passing them to the neural classifier for probability estimation, ensuring faithful explanations without relying on proxy models.

**Fig. 14:**
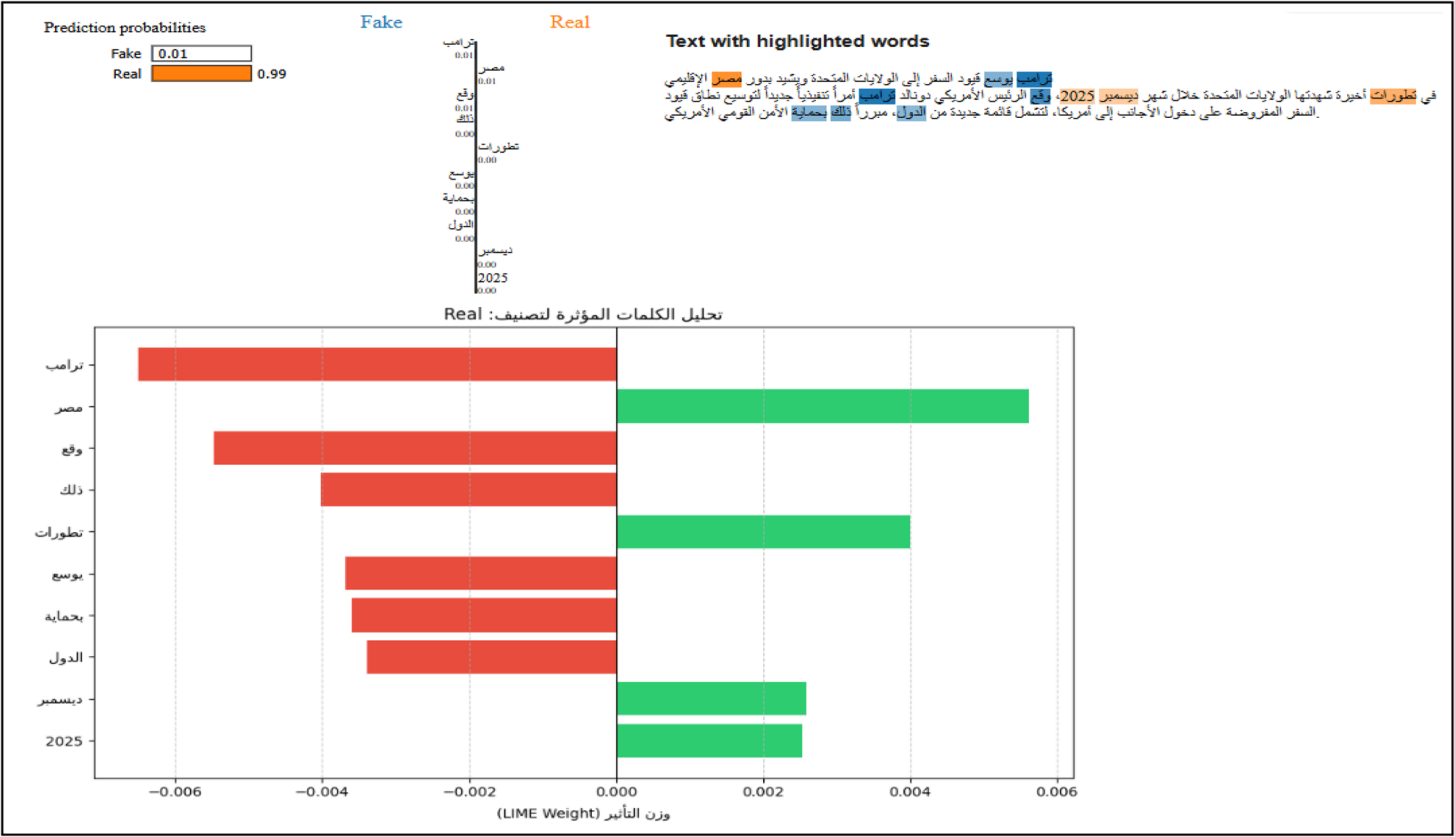
LIME Figure for Real News prediction (P=0.99).

**Fig. 15:**
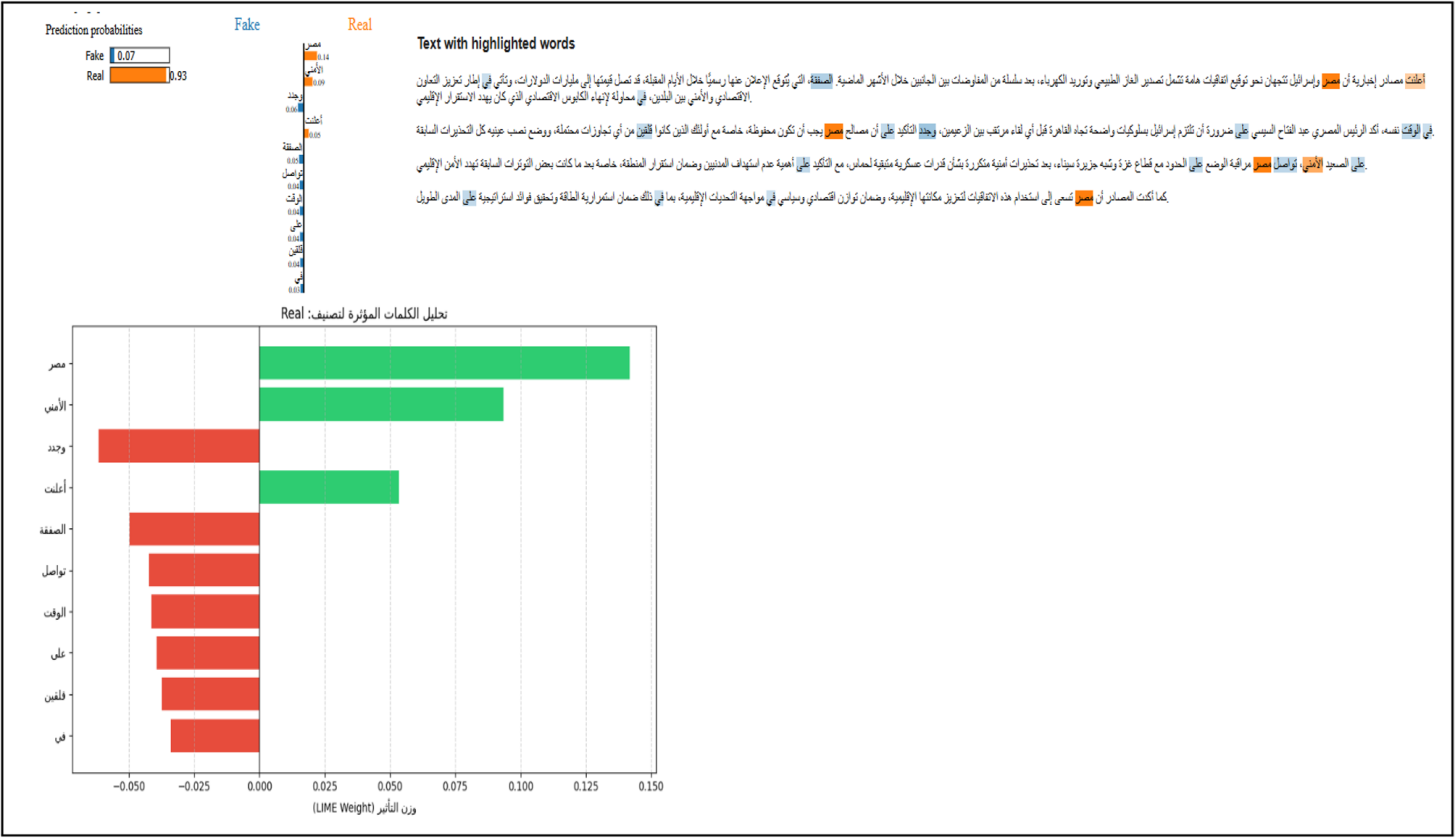
LIME Figure for Real News prediction (P=0.93).

**Fig. 16:**
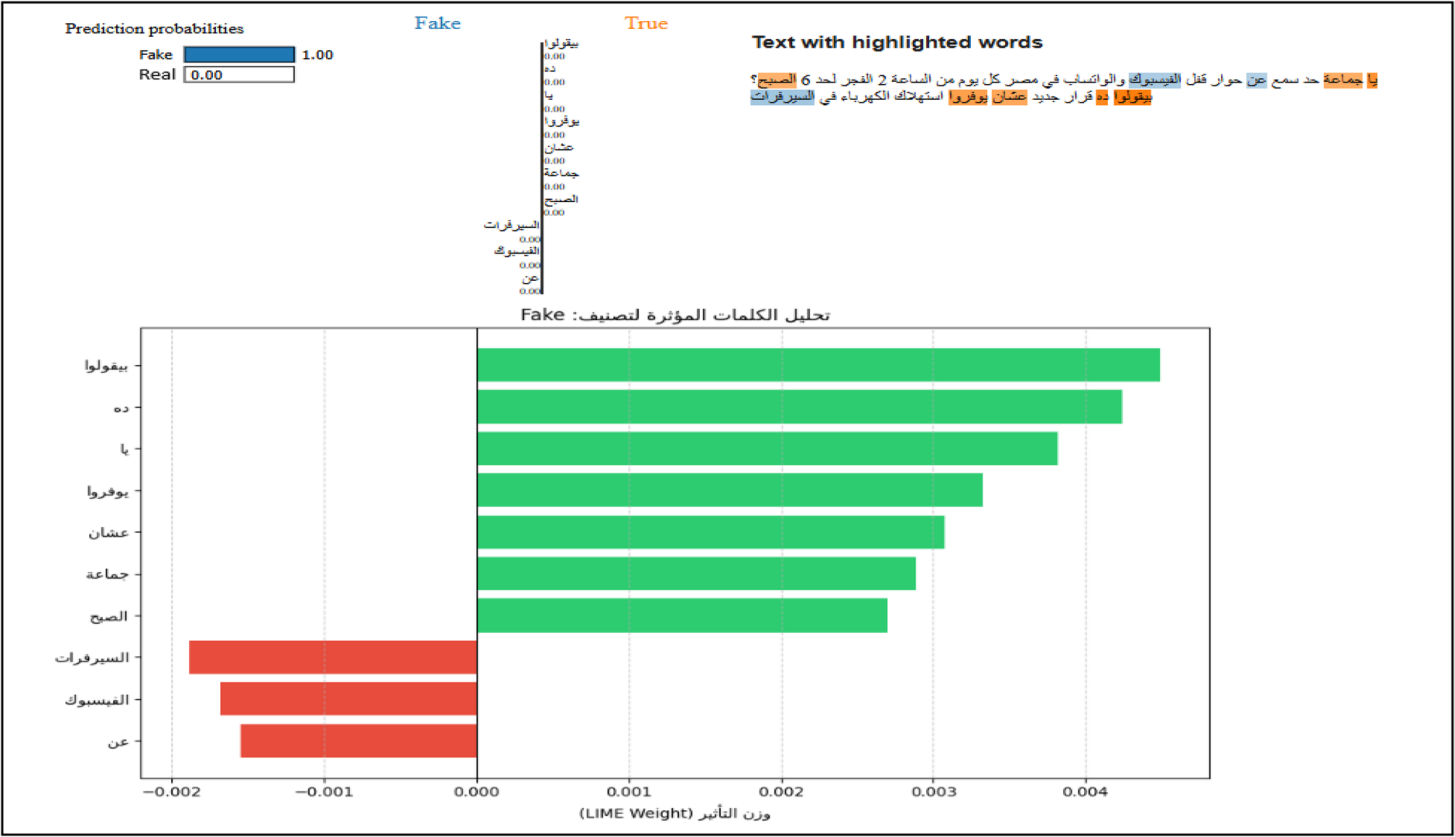
LIME Figure for Fake News prediction (P=1.00).

**Fig. 17:**
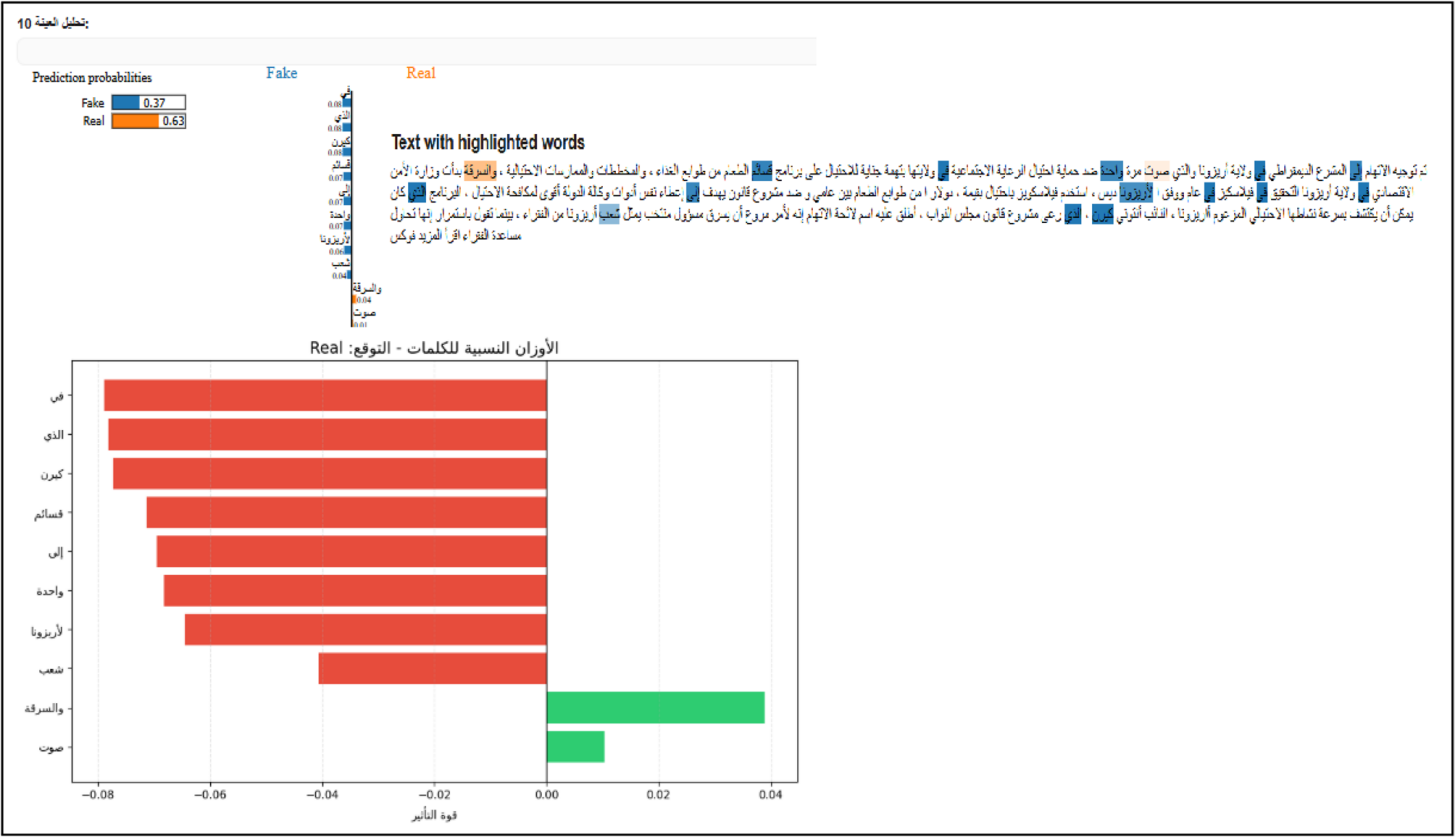
LIME Figure for Real News prediction (P=0.63).

- **Confidence in Authenticity:** Conversely, predictions classified as Real (Figs. 14 and 15) exhibit high confidence scores ranging from 93% to 99%, driven by the presence of formal linguistic anchors. Named entities such as “Masr” (Egypt),’Aalanat’ (Announced), and institutional references like “Al-Amny” (Security) along with specific temporal markers like ’December’ and ’2025’, served as primary positive contributors to the ’Real’ classification. This localized analysis demonstrates that the model has internalized associations between structured, official terminology and trustworthy reporting. These observations describe dataset-dependent tendencies rather than prescriptive linguistic rules, reinforcing the interpretability of the system without introducing bias assumptions.
- **Decoding Deceptive Dialect:** For instances predicted as Fake with high confidence (Fig. 16), LIME consistently assigns strong positive weights to informal Egyptian Arabic markers and conversational discourse cues. Tokens such as ’By’olou (they are saying), ’Dah’ (this), and ’Ya Gamaa’ (Hey people) emerge as influential indicators. These patterns reflect statistical correlations learned from the data and suggest that the model effectively distinguishes informal, rumor-driven narratives from professionally authored news content.
- **Case Analysis:** As illustrated in Fig. 17, the presence of specific named entities and formal institutional terms acts as a strong anchor for content authenticity. Even when certain informal expressions are present in the text (represented by red bars), the cumulative contribution of formal evidence (green bars) outweighs these signals, guiding the model toward a correct *Real* classification. This behavior demonstrates that the proposed system performs a weighted semantic synthesis of contextual cues rather than relying on isolated keyword matching.

### Comparison with State-of-the-Art methods

To validate the proposed model, we performed a two-tier comparison. First, we conducted a direct benchmarking against state-of-the-art Transformer architectures (AraBERT, MARBERTv2, and AraELECTRA) under the same experimental conditions as in Table 6. Second, we carried out a contextual comparison with performance metrics reported in the most recent literature, as presented in Table 10. Our model achieved an F1-score of 96.19%, accuracy of 95.52%, precision of 95.48%, and recall of 96.90%. Given the imbalanced nature of Arabic fake news datasets, F1-score provides a more reliable assessment than accuracy alone. Since prior studies rely on different datasets and experimental settings, the reported comparison serves as a contextual reference rather than a direct head-to-head evaluation.

- **Transformer-based benchmarks:** While AraBERT^3^ reported 92.3% accuracy on the AFND dataset, our model exceeds this by approximately 3.22%. Similarly, Koru et al.^23^ achieved 94.00% accuracy using a BERTurk + CNN hybrid; however, our CAMeLBERT-based framework demonstrates superior performance (95.52%) by leveraging embeddings specifically optimized for Modern Standard Arabic.
- **Hybrid architectures and stability:** Recent hybrid models like WaraBERT-V1^26^ achieved 93.83% accuracy. In contrast, the hybrid AraBERT + 2D-CNN approach by Othman et al.^25^ showed significant performance fluctuations across datasets, with F1-scores as low as 0.61. Our model’s stable F1-score of 96.19% highlights its robustness across diverse and multi-source Arabic news content.
- **Addressing class imbalance:** Unlike earlier deep learning models (CNN, LSTM, BERT-CNN hybrids) that generally range between 84% and 98% accuracy but often neglect class imbalance, our framework explicitly addresses this through algorithm-level Class Weighting. This ensures that the model maintains high precision and recall simultaneously, avoiding the decision bias and synthetic noise issues prevalent in many SOTA implementations.

The results indicate that our approach demonstrates competitive or superior performance compared to existing state-of-the-art methods, particularly in terms of F1-score, demonstrating its robustness and effectiveness in handling imbalanced Arabic news datasets. The integration of CAMeLBERT embeddings with deep neural network classifiers and algorithm-level class weighting is a key factor contributing to this superior performance. Furthermore, in addition to performance gains, the proposed framework differs from most prior studies by incorporating systematic interpretability analysis using LIME and SHAP.

**Table 10:** Comparison of the proposed model with selected state-of-the-art methods for Arabic fake news detection.

Reference	Dataset Used	Algorithms/Models Used	Model Results
Turki et al.^26^	AFND, AraNews	WaraBERT-V1 + BiLSTM (AFND), WaraBERT-V2 (AraNews)	Accuracy 93.83% (AFND), 81.25% (AraNews)
H.A.Bahri^3^	AFND	AraBERT (Transformer-based model)	Accuracy 92.3%, Macro F1-score 72%
Koru et al.^23^	TR_FaRe_News	BERTurk + CNN	Accuracy 94%
Othman et al.^25^	ANS, AraNews, Covid19Fakes	Hybrid AraBERT + 2D-CNN	Accuracy 71% (ANS), F1-score 0.6188 (ANS), 0.7837 (AraNews), 0.8009 (Covid19Fakes)
**Proposed Model**	Multi-source Arabic dataset	CAMeLBERT + Class Weighting + DNN	Accuracy 95.52%, F1-score 96.19%

## Discussion

The comprehensive experimental analysis conducted in Section “Experimental results” provides a deep understanding of the challenges and optimal solutions for Arabic fake news detection, validating the effectiveness of the proposed hybrid architecture. The discussion below offers an overall analysis of the models tested and highlights the strengths and inherent limitations of the proposed approach.

### Comparative model analysis and performance evolution

The proposed hybrid architecture combines CAMeLBERT embeddings with a deep neural network classifier, enabling the model to capture complex non-linear relationships that traditional linear classifiers may miss. This combination offers enhanced training efficiency. By employing CAMeLBERT as a fixed feature extractor with frozen weights, the model reduces computational overhead and accelerates convergence, making it highly suitable for practical deployment in real-world, resource-constrained environments. Furthermore, the model integrates algorithm-level Class Weighting to effectively handle class imbalance, ensuring that this computational efficiency does not come at the expense of predictive fairness for the minority class.

- **The superiority of contextualized representations:** The significant performance leap from TF-IDF-based models (90.97% accuracy) to CAMeLBERT-based models (95.52% accuracy) underscores the necessity of deep contextual understanding in Arabic NLP. The stability analysis (Section "Training and stability analysis of baseline model (TF-IDF + NN)") revealed severe overfitting for TF-IDF, indicating that frequency-based feature vectors were insufficient to generalize across the linguistic complexity of Arabic news. While traditional frequency-based methods rely on lexical overlap, they fail to distinguish between the subtle semantic nuances of deceptive and legitimate news. Transformer models like CAMeLBERT, AraBERT, and MARABERT provided a significant performance boost due to their ability to capture deep semantic and contextual cues in Arabic text. CAMeLBERT’s specific focus on Modern Standard Arabic, the widely used formal language across the Arab world, provided a robust semantic baseline for detecting Arabic fake news. The high recall (96.90%) suggests that the self-attention mechanism effectively identifies structural patterns common in misinformation, such as hyperbolic language or lack of institutional referencing.
- **Algorithmic calibration vs. data resampling:** A key finding of this study is that addressing class imbalance at the algorithmic level through Class Weighting is more effective than data-level interventions such as SMOTE or undersampling. Unlike SMOTE, which may generate synthetic embeddings lacking semantic coherence in Arabic, Class Weighting preserves the original distribution while penalizing errors in the minority class, maintaining data integrity. Furthermore, the implementation of Class Weighting successfully reduced False Positives by 51.2% compared to the baseline CAMeLBERT model (FP from 82 to 40). These results demonstrate that algorithm-level calibration not only preserves semantic fidelity but also ensures a more reliable and balanced predictive performance in imbalanced Arabic fake news datasets. This aligns with prior studies conducted in English and other languages, which similarly report the superiority of transformer-based architectures over traditional approaches, thereby extending this empirical evidence to the Arabic language domain. It is noteworthy that while some resampling techniques yielded a slightly higher AUC-ROC, the Class Weighting approach was selected as the optimal solution. In the domain of Arabic fake news detection, the F1-score provides a more reliable metric for operational performance as it directly accounts for the precision-recall trade-off at a specific decision threshold. While AUC-ROC measures the general discriminative potential across all thresholds, the Class Weighting model’s ability to minimize total misclassifications (achieving FN=27 vs. FP=40) proves its practical effectiveness and robustness in real-world verification scenarios.
- **Interpretability as a proxy for robustness:** The integration of SHAP and LIME provides more than just transparency; it validates the model’s decision-making process. The SHAP analysis confirmed that the classifier effectively exploits a wide spectrum of the 768-dimensional embedding space, indicating that the neural network has learned complex, non-linear representations of ”deceptiveness.” Furthermore, the LIME analysis revealed a fascinating linguistic phenomenon: the model’s reliance on informal Arabic dialect markers (e.g., ’Ya Gamaa’, ’Ashan’) as indicators of fake news, versus formal institutional anchors for real news. This suggests that the model has implicitly learned to distinguish the ”source authority” based on linguistic style–a capability that frequency-based models lack. This aligns with sociolinguistic theories that suggest misinformation in the Arab world often propagates through informal, conversational channels rather than structured media outlets.

### Real-world applications and limitations

The success of the proposed CAMeLBERT + Class Weighting + NN model has several important implications for real-world deployment. The framework was explicitly designed with practical scalability and robustness in mind, making it suitable for critical decision-making environments where accuracy and reliability are paramount.

One key application of the proposed approach lies in fact-checking and early warning systems. The model can be integrated into automated fact-checking platforms to assist journalists and researchers in prioritizing news articles for verification. Additionally, when linked with social media APIs, the system can function as an early-warning mechanism by issuing real-time alerts upon detecting suspicious or rapidly spreading content. Furthermore, the framework can be employed in social media and institutional monitoring tools to help organizations and public institutions mitigate the rapid dissemination of disinformation. By identifying potentially misleading content at an early stage, such systems can reduce the societal impact of false narratives before they gain significant traction. These applications highlight the importance of maintaining a balanced trade-off between False Positives and False Negatives. The final optimized model successfully achieves this balance, ensuring that harmful misinformation is effectively flagged while minimizing unnecessary alerts for legitimate content.

Despite the efficiency of the training phase, relying on the full CAMeLBERT architecture during inference introduces significant memory usage and latency. This poses challenges for deployment on edge devices and resource-constrained platforms. Future research into model compression and knowledge distillation techniques will be essential to reduce the inference footprint while preserving high detection accuracy.

Moreover, the rapidly evolving nature of Arabic language usage in online environments presents an ongoing challenge. The emergence of new slang, linguistic blending, and code-switching phenomena necessitates frequent model updates and retraining to maintain detection accuracy over time. Without such adaptation, performance may degrade as online discourse continues to evolve. While the proposed model demonstrates strong performance on Modern Standard Arabic textual data, its generalization remains limited with respect to dialectal Arabic and multimodal content. Since fake news on digital platforms is often expressed in dialectal forms and accompanied by images or videos, extending the framework to support multimodal inputs and broader linguistic coverage would significantly enhance its real-world applicability. In addition to these technical limitations, ethical considerations related to data privacy and algorithmic fairness must be carefully addressed prior to large-scale deployment, particularly in sensitive public and institutional contexts.

## Conclusion and future work

This study addressed the critical challenge of detecting Arabic fake news by systematically evaluating traditional machine learning models, neural network baselines, and state-of-the-art Transformer models under class-imbalanced conditions. While Naïve Bayes, Decision Trees, and KNN applied on TF-IDF features achieved moderate performance, initial experiments identified the neural network as the strongest baseline, achieving an F1-score of 92.20% and an accuracy of 90.97%. The integration of CAMeLBERT contextual embeddings as a fixed feature extractor further enhanced performance, achieving an F1-score of 95.05% and an accuracy of 93.98%, consistently outperforming other Transformer variants, including AraBERT and MARBERTv2. The core contribution of this research lies in demonstrating that algorithm-level strategies, specifically Class Weighting, provide a more stable and robust solution than data-level resampling (SMOTE, oversampling, and undersampling), which often introduce synthetic noise or lead to information loss. Our final optimized model, CAMeLBERT + Class Weighting + DNN, achieved peak accuracy of 95.52% and an F1-score of 96.19%, providing an optimal balance between precision and recall. Furthermore, this study addressed the ”black-box” nature of deep learning by incorporating SHAP and LIME interpretability analysis, offering clear evidence-based insights into how the model identifies misinformation markers in Arabic text. Additionally, we compiled a comprehensive multi-source Arabic news dataset to support robust model evaluation, highlighting the effectiveness of the proposed approach in handling diverse and imbalanced Arabic news content. Looking ahead, future work will focus on evaluating the model’s generalizability across diverse news domains (e.g., sports, health, and finance) and various Arabic dialects. It can also be extended to analyze multimodal content (text, images, and videos) using fusion techniques. Additionally, incorporating external knowledge bases and real-time verification APIs could enhance the veracity assessment of emerging news trends, supporting real-time fact-checking applications. In conclusion, this research provides a high-performance, transparent, and scalable framework that advances the state-of-the-art in Arabic fake news detection, offering a notable contribution toward preserving information integrity in the digital Arabic landscape.

## Data Availability

the dataset generated and analyzed during the current study isn’t publicly available and is available from the corresponding author on reasonable request
